# The CXCR4 antagonist R54 targets epithelial-mesenchymal transition (EMT) in human ovarian cancer cells

**DOI:** 10.1371/journal.pone.0314735

**Published:** 2024-12-19

**Authors:** Daniela Russo, Anna Spina, Luigi Portella, Anna Maria Bello, Francesca Galdiero, Anna Maria Trotta, Caterina Ieranò, Giuseppina Rea, Sabrina Chiara Cecere, Elisabetta Coppola, Salvatore Di Maro, Sandro Pignata, Daniela Califano, Stefania Scala

**Affiliations:** 1 Microenvironment Molecular Targets, Istituto Nazionale Tumori-IRCCS-Fondazione "G. Pascale", Naples, Italy; 2 Uro-Gynecologic Oncology Unit, Istituto Nazionale Tumori IRCCS-Fondazione "G. Pascale", Naples, Italy; 3 Department of Environmental, Biological and Pharmaceutical Sciences and Technologies, University of Campania "Luigi Vanvitelli", Caserta, Italy; Universita degli Studi della Campania Luigi Vanvitelli, ITALY

## Abstract

The axis CXCL12-CXCR4 is highly expressed in ovarian cancer where contributes to disease progression. Aim of the work was to evaluate the effect of the newly developed CXCR4 antagonist R54 on human ovarian cancer cells aggressiveness. CXCL12-CXCR4 axis was evaluated in human ovarian cancer cells through proliferation, migration and signaling CXCL12-dependents. Epithelial to mesenchymal transition (EMT) was analyzed through *E-CADHERIN*, *N-CADHERIN*, *VIMENTIN*, *SNAIL1* and *ΒETA-CATENIN* by qRT-PCR, immunofluorescence and immunoblotting. R54 inhibited ovarian cancer cells proliferation and migration CXCL12-induced. Moreover, R54 inhibited CXCL12 dependent pERK1/2 and pAKT and reversed the CXCL12 induced EMT in ovarian cancer cells. Targeting CXCR4 with the new antagonist R54 consistently reverted the mesenchymal transition in human ovarian cancer cells reducing migratory and chemoresistance features.

## 1. Introduction

Epithelial Ovarian Cancer (EOC) is the eighth most common cause of cancer death for women worldwide [1]. The majority of ovarian cancers are diagnosed in advanced stages [2] and achieve complete response (CR) with front-line platinum-based chemotherapy [3]. However, the survival rate of patients remains low due to drug resistance and disease relapse [4]. Epithelial to mesenchymal transition (EMT) [5, 6] plays a central role in EOC progression and chemoresistance [7] with chemokines and related receptors acting as EMT inducers [8]. CXCL12 and CXCR4 expression in EOC is associated with higher grades, increased risk of recurrences, and poor survival [9, 10]. The CXCR4 ligand CXCL12 also insists on CXCR7 that is an atypical chemokine receptor (ACKR) which binds CXCL12 and CXCL11. Unlike CXCR4, CXCR7 preferentially transmits signals through the non-classical β-arrestin pathway. CXCR7 modulates CXCR4 activity by scavenging CXCL12 due to its higher ligand affinity than CXCR4 and forming heterodimers with CXCR4 altering its downstream signaling [11], Recently, the axis CXCR4-CXCL12-CXCR7 was evaluated on 308 EOC samples showing that the majority expressed CXCR4, CXCL12 and CXCR7. Although CXCL12 was not prognostic, its epithelial expression identified high-risk FIGO stage III patients for progression free survival [12]. CXCR4/WNT/BETA-CATENIN induction in ovarian cancer cells leads to cisplatin resistance [13] and *in vivo* CXCR4 targeting inhibited intraperitoneal dissemination [14]. CXCR4 antagonists are in different phases of development [15] among those a new family of CXCR4 antagonists was developed with Peptide R54 (R54) as lead compound [16, 17]. Aim of the study was to evaluate the efficacy of R54 on CXCR4-CXCL12 axis in ovarian cancer cells to provide a new tool for ovarian cancer therapy.

## 2. Materials and methods

### 2.1 Cell culture

Human OVCAR8, IGROV1, A2780, MDAH2274, TOV112D, SKOV3, OVCAR3, OVCAR4 and OVCAR5 were cultured in RPMI-1640 medium supplemented with 10% FBS, 1% penicillin/streptomycin and 1% L-Glutamine at 37°C in a humidified incubator with 5% CO2, while CAOV3 were cultured in Dulbecco’s modified Eagle medium (DMEM) supplemented with 10% FBS, 1% penicillin/streptomycin and 1% L-Glutamine at 37°C in a humidified incubator with 5% CO2. IGROV1 cells were knocked out for *CXCR4* (IGROV1 CXCR4-KO) through the *CXCR4* Human Gene Knockout Kit (CRISPR) (OriGene #KN402069). In details, cells were seeded in a 6-well plate at a density of 2,2 x 10^5^ cells/well in 2 mL of RPMI 1640 Medium supplemented with 10% FBS. After 24 hours the cells were transfected using 3,75μL of Lipofectamine 3000 Transfection Reagent (Thermo Fisher Scientific), 1 μg of Cas9/gRNA vector and 1 μg of the green fluorescent protein–puromycin linear donor vector. Puromycin was then added at a final concentration of 3 μg/ml for 15 days before isolation of puromycin-resistant cell clones. CXCR4 expression was verified by flow cytometry and by Real time-qPCR. Clone #9 was selected to conduct experiments.

### 2.2 Growth curve

CAOV3, OVCAR8 and IGROV1 were evaluated for cell growth in presence of CXCL12/chemotherapeutics/R54 and combinations. 1x10^5^ CAOV3, 4x10^4^ OVCAR8 and 1x10^5^ IGROV1 cells were seeded in 2 ml serum free media in a 6 well plate and allowed to attach for 16 hours. The following day cancer cells were treated with 100 ng/mL CXCL12 plus/minus 100 nM R54 and 5 μM, 2 μM and 250 nM of cisplatin and 1 nM, 3 nM and 2 nM of paclitaxel for CAOV3, OVCAR8 and IGROV1 respectively. Each experimental condition was run in duplicate. The one-way ANOVA test was used to compare the means between experimental conditions and to identify statistically significant differences. Data are expressed as the average of at least two independent experiments ±SD.

### 2.3 Flow cytometry

EOC cells at 50–70% confluence were detached with 2 mmol/L EDTA in PBS, washed twice in ice-cold PBS, suspended in 1 ml of complete culture medium and incubated for 2 hours at 37°C and 5% CO2. Cells were then washed in ice-cold PBS and stained in 98 μl of staining buffer plus 2 μl of anti-human CXCR4-PE conjugated mouse IgG2a (Catalog Number: FAB170P R/D Systems, Minneapolis, MN, USA) and anti-human CXCR7-RDC-1 APC conjugated mouse IgG1 (Catalog Number: FAB4227A R/D Systems, Minneapolis, MN, USA) for 45 min at 4°C in the dark. Cells were then washed in ice-cold PBS and suspended in 100 μl of staining buffer. Samples were analysed with a FACS ARIA III cytometer (BD Bioscience) and FlowJo software (BD Bioscience). Appropriate fluorochrome-conjugated isotype matched antibodies were used as control to establish background.

### 2.4 Immunofluorescence

CAOV3, OVCAR8 and IGROV1 cells 2x10^4^ were plated on 15 mm glass coverslips in 24 well plate and allowed to attach for 16 hours. The following day cancer cells were fixed with 200 μL 4% PFA at 4°C for 10 min, washed three times with PBS and blocked with PBS- 5% (w/v) BSA (Fraction V, Sigma-Aldrich, St. Louis, MO, USA) for 1 hour at room temperature. CXCR4 1:300 (cat n. ab124824, ABCAM), E-CADHERIN 1:500 (cat n. 610182, BD Bioscience, vial concentration 250 μg/ml) and VIMENTIN 1:500 (cat n. ab92547, ABCAM, vial concentration 0.232–0.268 mg/mL-batch-dependent) were incubated at 4°C for 16h in 200 μL PBS-5% BSA. After washing with PBS, incubation with 1:300 Alexa-488 conjugated secondary antibodies (cat. n. 111-545-003 anti-rabbit for CXCR4 and cat. n. 115-545-005 anti-mouse for CXCR7, Jackson Immunoresearch Laboratories, antibody concentration 1,5 mg/ml) in 200 μL PBS 5% BSA was performed at room temperature for 1 hour in the dark. 200 μL of DAPI staining solution was added to the coverslip for 5 minutes. Coverslips were mounted on glass slides with Moviol. All images were captured with 40X objective.

### 2.5 qRT-PCR

Total RNA was isolated using RNeasy Mini Kit (cat. n.74104, Qiagen). One microgram of RNA was retro transcripted using QuantiTect Reverse Transcription kit (Cat. n. 205311, Qiagen) according to manufacture. 20 ng of cDNA was used to perform qRT-PCR) using SsoAdvanced Universal SYBR Green Supermix (cat. n. 1725271, Bio-rad). Human specific primers were designed for:

- *CXCR4* (forward: 5′-TGGGTGGTTGTGTTCCAGTTT-3′ and reverse: 5′-ATGCAATAGCAGGACAGGATGA-3′);
- *CXCR7* (forward: 5′-GATTGCCCGCCTCAGAAC-3′ and reverse: 5′-GCAGGACGCTTTTGTTGG-3′);
- *CXCL12* (forward: 5′-TGTGGCACTCAGATACCGACT-3′ and reverse: 5′-CCCACAGAGCCAATCACT-3′);
- *E-CADHERIN* (forward: 5′-TGAGTGTCCCCCGGTATCTT-3′ and reverse: 5′-CAGTATCAGCCGCTTTCAGA-3′);
- *VIMENTIN* (forward: 5′-GAGAGGAAGCCGAAAACACC-3′ and reverse: 5′-GCGTTCAAGGTCAAGACGTG-3′);
- *N-CADHERIN* (forward: 5′-CCATCATTGCCATCCTGCTC-3′ and reverse: 5′-CGGCGTTTCATCCATACCAC-3′);
- *SNAIL1* (forward: 5′-GACCCCAATCGGAAGCCTAA-3′ and reverse: 5′-GTAGGGCTGCTGGAAGGTAA-3′).
- *GUSB2* (forward: 5′-AGCCAGTTCCTCATCAATGG-3′ and reverse: 5′-GGTAGTGGCTGGTACGGAAA-3′).
- *Β2M* (forward: 5′-CATTCCTGAAGCTGACAGCATTC-3′ and reverse: 5′-TGCTGGATGACGTGAGTAAAC-3′).
- *α-TUBULIN* (forward: 5′-CCGGGCTGTGTTTGTAGACT-3′ and reverse: 5′-GATCTCCTTGCCAATGGTGTA-3′).

Relative gene expression was compared to *GUSB* or a median of *GUSB* and *B2M* in IGROV1 and CAOV3, a median of *GUSB* and *α-TUBULIN* in OVCAR8 as endogenous control. Relative expression of the target genes was determined using the 2^ −ΔCq^ or 2^ − ΔΔCq^ method. Each sample was run in triplicate and the data presented as means ± SD of the 2^ − ΔCq^ or 2^ − ΔΔCq^ values derived from two biological replicates.

### 2.6 Western blotting

Cells were lysed with a lysis buffer containing 10 mM TrisHCl pH 8.0, 150 mM NaCl, 1% NP40, 0,4 mM EDTA, protease inhibitors (Complete Tablets-EDTA-free, Roche) and phosphatase inhibitors (2 mM NaOv and 10 mM NaF). Each sample was run on two gels in parallel; after protein transfer, molecular weight marker were labelled with a pen and nitrocellulose membranes cut according to the molecular weight of investigated protein. Blots were incubated with 1:1000 mouse anti-CXCR4 antibody (cat n. 60042-1-Ig Proteintech), 1:1000 rabbit polyclonal anti-phospho-p44/42 MAPK (ERK1/2) (Thr202/Tyr204) antibody (cat n. 9101 Cell Signaling Technology), 1:1000 rabbit polyclonal anti-p44/42 MAPK (ERK1/2) antibody (cat n. 9102 Cell Signaling Technology), 1:1000 rabbit polyclonal anti-phospho-AKT (Ser 473) antibody (cat n. 9271 Cell Signaling Technology), 1:1000 rabbit polyclonal anti-AKT antibody (cat n. 9272 Cell Signaling Technology), 1:1000 rabbit polyclonal anti-phospho-p38 MAPK (Thr180/Tyr182) (D3F9) XP antibody (cat n. 4511 Cell Signaling Technology), 1:1000 rabbit polyclonal anti-p38 MAPK antibody (cat n. 9212 Cell Signaling Technology), 1:1000 mouse monoclonal anti-RAC 1/2/3 Antibody G-2 (cat n. sc-514583 200 μg/ml Santa Cruz Biotechnology, INC.), 1:1000 anti-E-CADHERIN (24E10) rabbit mAb (cat n. 3195 Cell Signaling Technology), 1:1000 anti-VIMENTIN (D21H3) XP rabbit mAb (cat n. 5741 Cell Signaling Technology), 1:1000 anti-BETA-CATENIN (D10A8) XP rabbit mAb (cat n. 480 Cell Signaling Technology), 1:20000 monoclonal mouse anti–α-tubulin antibody (cat n. T9026 Sigma-Aldrich) in TBS containing 5% BSA and 0,01% Sodium Azide overnight at 4°C. Anti-mouse (Goat anti-Mouse IgG H+L HRP Conjugated cat n. 62–6520, Invitrogen) or anti-rabbit IgG (Goat Anti-Rabbit IgG H+L HRP Conjugated cat n. 1706515, Bio-rad) were used as secondary antibodies and the signal was revealed through chemo luminescent detection kit (Pierce ECL Western Blotting Substrate cat n. 32106, Thermo Scientific).

### 2.7 Cell migration

Cell migration was assessed in 24-well Transwell chambers (Corning Inc., Corning, NY) using inserts with an 8-μm pore membrane. Membranes were pre-coated with 10 g/ml collagen (human collagen type I, cat. n. C5533 Sigma Aldrich) and 10 g/ml fibronectin (cat. n.F0895 Sigma Aldrich). CAOV3 OVCAR8, IGROV1 and IGROV1 CXCR4-KO (2.0 × 10^5^ cells in 100 μL/well) were placed in the upper chamber of the insert in migration medium (RPMI supplemented with 0.5% BSA for OVCAR8, IGROV1 and IGROV1 CXCR4-KO and DMEM supplemented with 0.5% BSA for CAOV3) in presence of R54 100 nM; cells were allowed to migrate toward migration medium containing 0,5% BSA +/- CXCL12 100 ng/mL or CXCL11 100 ng/mL in the lower well for 16 hours at 37°C in a humidified incubator in 5% CO2. The following day, non-migrating cells were removed from the upper chamber using a cotton swab, and the cells that had migrated to the lower surface of the insert were fixed in 4% (w/v) PFA in PBS and stained for 15 min with DAPI (1:25.000, sc-3598 Santa Cruz). Cells were visualized with a fluorescent microscope (Carl Zeiss, Axio Scope.A1) and counted (cells in 10 randomly chosen fields). Migration index is the ratio between the number of cells migrating toward 100 ng/ml CXCL12/CXCL11 containing medium and the number of cells migrating toward 0.5% BSA medium (n. of cells migrating toward CXCL12 or CXCL11 medium/n. of cells migrating toward BSA medium). The one-way ANOVA test was used to compare the means between experimental conditions and to identify statistically significant differences.

## 3. Results

### 3.1 Human ovarian cancer cells express functional CXCR4-CXCL12-CXCR7 axis

**Fig 1:**
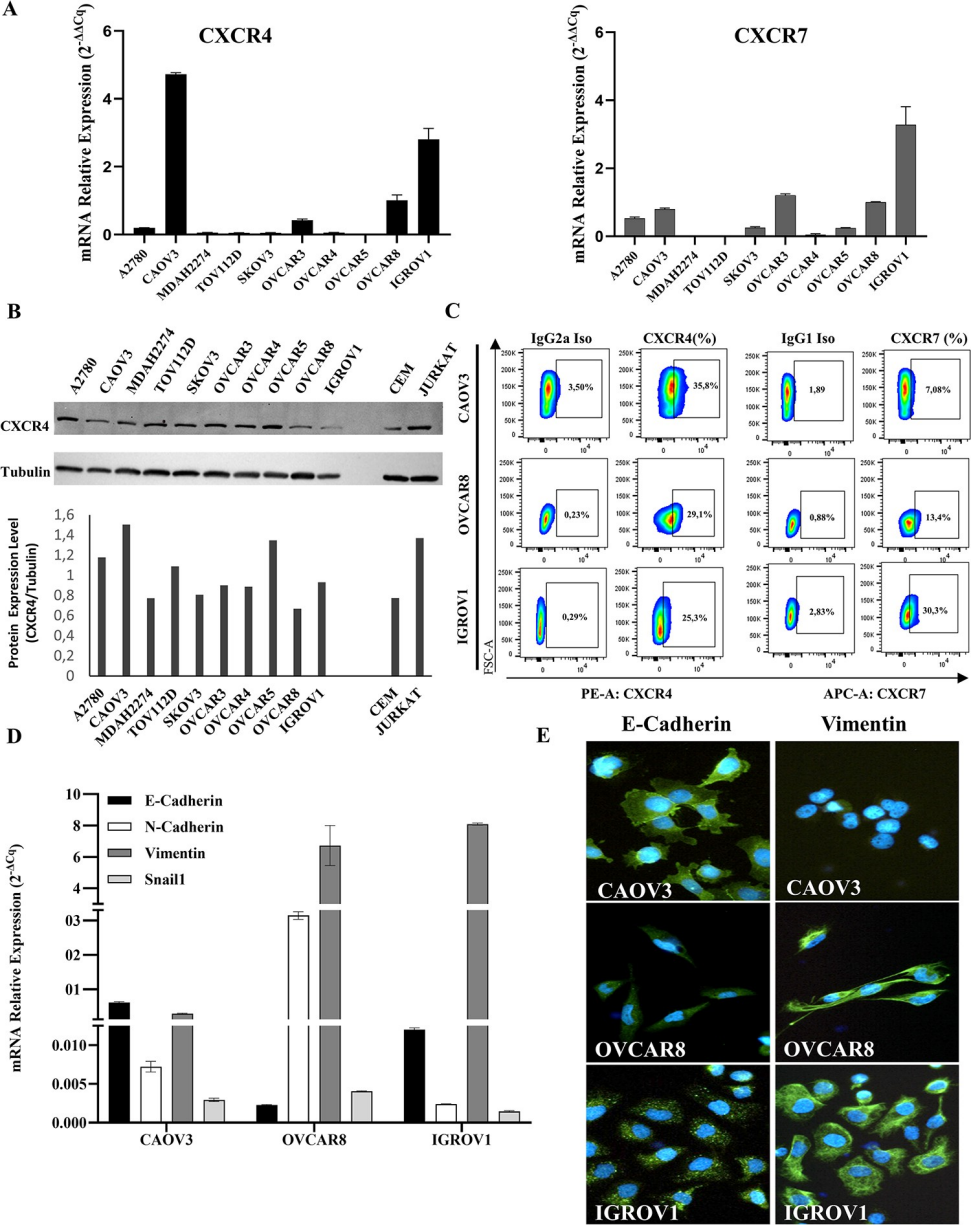
Human ovarian cancer cell lines express CXCR4-CXCL12-CXCR7 axis and EMT markers. (A) qRT-PCR for *CXCR4* and *CXCR7* in human ovarian cancer cells. Gene expression was calculated using 2^-ΔΔCq^ method (normalized to *GUSB)*. (B) Western Blotting for CXCR4 in human ovarian cancer cells; CEM and Jurkat cells were used as positive controls. (C) Flow cytometry for CXCR4 and CXCR7 in CAOV3, OVCAR8, IGROV1. (D) qRT-PCR for E*-CADHERIN*, N-*CADHERIN*, V*IMENTIN* and S*NAIL*1 in CAOV3, OVCAR8 and IGROV1 cell lines. Gene expression was calculated using 2^-ΔCq^ method (normalized to the mean of *GUSB* and *α-TUBULIN* in OVCAR8 and to the mean of *GUSB* and *B2M* in IGROV1 and CAOV3). (E) Immunofluorescence for E-CADHERIN and VIMENTIN in CAOV3, OVCAR8 and IGROV1 cell lines.

*CXCR4*, *CXCR7* and *CXCL12* expression was evaluated in human ovarian cancer cells A2780, CAOV3, MDAH2274, TOV112D, SKOV3, OVCAR3, OVCAR4, OVCAR5, OVCAR8 and IGROV1 (Fig 1A and S1A Fig). As reported, CAOV3, OVCAR3, OVCAR8 and IGROV1 expressed high *CXCR4* (Fig 1A), IGROV1 displayed high *CXCR4/CXCR7* (Fig 1A), while OVCAR4 expressed *CXCL12* (S1A Fig). To better characterize the expression of CXCR4, Western Blot was performed in all tested ovarian cancer cell lines. As shown in Fig 1B the evaluated human ovarian cancer cells express CXCR4. In particular, A2780, CAOV3 and OVCAR5 overexpress CXCR4 as compared to Jurkat and CEM, human lymphoblastic cells, high CXCR4 expressing cells. Thus, we focused on CAOV3, OVCAR8 and IGROV1 for further characterization. CXCR4 and CXCR7 were further detected on cell membrane in CAOV3, OVCAR8 and IGROV1 in Fig 1C and S1B Fig (only CXCR4); CXCR4 was surface expressed in CAOV3>OVCAR8>IGROV1 (% CXCR4 expression: 35,8% in CAOV3, 29,1% in OVCAR8 and 25,3% in IGROV1) while CXCR7 was surface expressed in IGROV1>OVCAR8>CAOV3 (% CXCR7 expression: 30,3% in IGROV1, 13,3% in OVCAR8 and 7,08 in CAOV3). To assess the EMT status, *E-CADHERIN*, *N-CADHERIN*, *VIMENTIN* and *SNAIL1* were assessed by qRT-PCR (Fig 1D) and E-CADHERIN and VIMENTIN also evaluated by immunofluorescence (Fig 1E). CAOV3 expressed *E-CADHERIN*, low *N-CADHERIN* and *VIMENTIN*, OVCAR8 mainly expressed the mesenchymal markers *N-CADHERIN* and *VIMENTIN* while IGROV1 expressed low *E-CADHERIN* and high level of *VIMENTIN*; SNAIL1 was low expressed in all assessed cell lines (Fig 1D). CAOV3 were the most epithelial cells (E-CADHERIN positive and VIMENTIN negative), OVCAR8 the most mesenchymal cells (high VIMENTIN), while IGROV1 co-expressed E-CADHERIN and VIMENTIN indicating an intermediate phenotype (Fig 1D and 1E).

### 3.2 R54 impairs CXCR4 mediated migration in ovarian cancer cells

**Fig 2:**
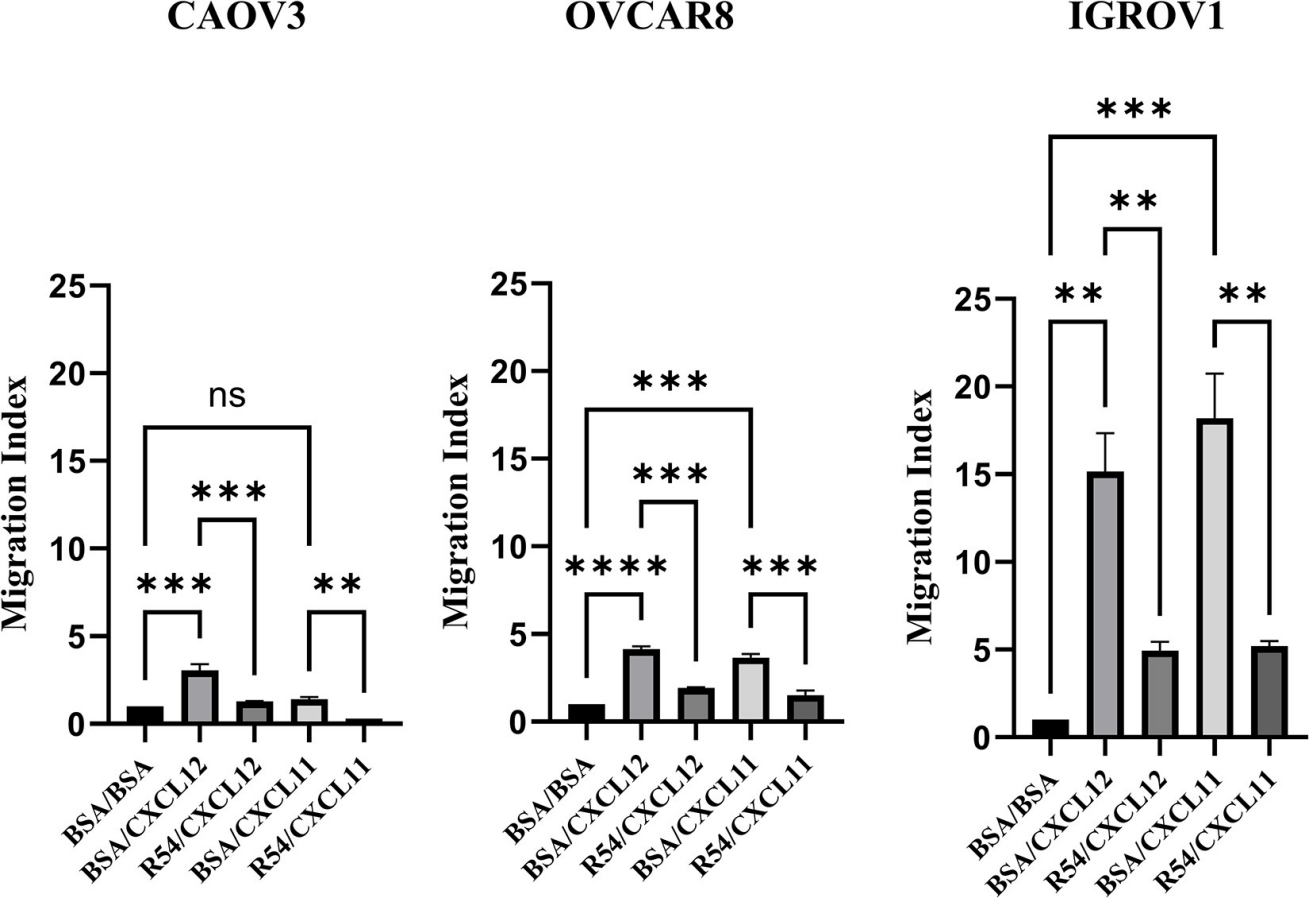
CXCL12 induced CAOV3, OVCAR8 and IGROV1 migration, while R54 impaired it. CAOV3, OVCAR8, IGROV1 cells migration (20.000 cell/well) was evaluated through Transwell filter; cells were allowed to migrate toward 100 ng/ml CXCL12 or CXCL11 +/- R54 100 nM for 16 hours and then counted. ** denote p < 0,01,*** denote p < 0,001, **** denote p < 0,0001.

To evaluate CXCR4 and CXCR7 function, CXCL12/CXCL11 dependent migration was conducted in the presence of the new CXCR4 antagonist R54 (Fig 2). In Fig 2, IGROV1 robustly migrated toward CXCL12 (15 fold as compared to BSA, p<0,01) while OVCAR8 and CAOV3 migration index was respectively 4 and 3 fold higher than BSA (p<0,0001 for OVCAR8 and p<0,01 for CAOV3). R54 significantly impaired CXCL12-mediated migration in CAOV3 (2,5 fold lower than CXCL12, p<0,01), OVCAR8 (2,1 fold lower than CXCL12, p<0,001) and IGROV1 (3 fold lower than CXCL12, p<0,01). To confirm the CXCR4-dependent migration, CXCR4 gene was knocked out in IGROV1 cells (S2A and S2B Fig). IGROV1 CXCR4-KO Clone #9 cells did not significantly migrate toward CXCL12 (CXCL12-migration index for IGROV1 WT 35.3 (p<0,0001) vs 1.9 for IGROV1 CXCR4-KO (p<0,1) and R54 had no impact (S2C Fig). CXCL11- mediated migration reflects CXCR7 expression in ovarian cancer cell lines. IGROV1, higher CXCR7 expressing cell line, clearly migrated toward CXCL11 (18 fold as compared to BSA, p<0,001), OVCAR8 migration index toward CXCL11 was 3,6 fold higher than BSA (p<0,001) and CAOV3 (lower CXCR7 expressing) CXCL11-migration index increase was not significant (Fig 2). R54 impaired CXCL11-mediated migration particularly in IGROV1 (3 fold lower than CXCL11, p<0,01) and in OVCAR8 (2 fold lower than CXCL11, p<0,001) (Fig 2).

### 3.3 R54 inhibits CXCL12-mediated mesenchymal transition in human ovarian cancer cells

**Fig 3:**
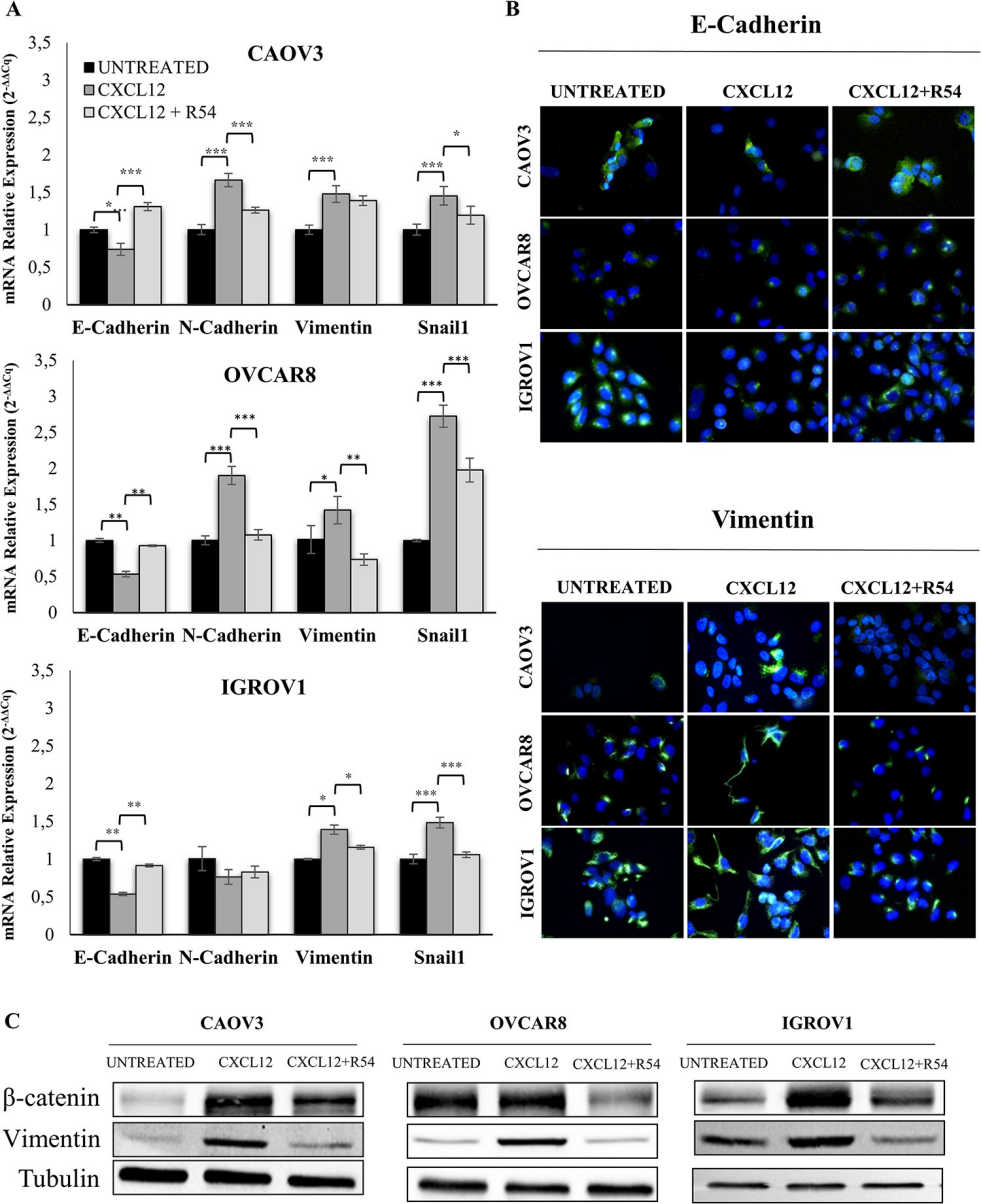
R54 inhibited epithelial-mesenchymal transition CXCR4 mediated. (A) qRT-PCR for EMT markers in human ovarian cancer cells were performed after stimulation with CXCL12 (100 ng/mL ± R54 100 nM). Gene expression was calculated using 2^-ΔΔCq^ method (normalized to the mean of *GUSB* and *α-TUBULIN* in OVCAR8 and to the mean of *GUSB* and *B2M* in IGROV1 and CAOV3) as the fold increase compared with untreated cells. Data are presented as mean values ± Standard Deviation. *denote p<0,05, ** denote p<0,005 *** denote p<0,0005; (B) Immunofluorescence for E-CADHERIN and VIMENTIN after treatment with CXCL12 +/- R54 in CAOV3, OVCAR8 and IGROV1 cells; (C) Western Blots for BETA-CATENIN and VIMENTIN after 24 hours of stimulation with CXCL12 100 ng/mL ± R54 100 nM in CAOV3, OVCAR8 and IGROV1 cells.

CXCL12 was reported to induce EMT in ovarian cancer [18]. Thus, the impact of R54 was evaluated on EMT related proteins. As observed in Fig 3A, CXCL12 reduced *E-CADHERIN* and upregulated the mesenchymal *VIMENTIN* and *SNAIL1* expression. Moreover, CXCL12 induced *N-CADHERIN* in CAOV3 and OVCAR8 cell lines (Fig 3A). R54 prevented the mesenchymal transition increasing *E-CADHERIN* and attenuating the mesenchymal markers expression (Fig 3A).To evaluate the role of CXCR7 in EMT, CXCL11 mediated EMT genes expression was analysed in ovarian cancer cells plus R54. As showed in S3A Fig, VIMENTIN expression increased in all cell lines and R54 reverted the induction. As R54 does not interact with CXCR7 [17], the effect of R54 in combination with CXCL11 may derive from CXCR4 heterodimerized with CXCR7 receptor. In IGROV1-CXCR4 KO cells, CXCL12 and R54 had negligible effect (S4A Fig). As shown in Fig 3B and S5 Fig, R54 reverted the effects of CXCL12 increasing in E-CADHERIN and reducing VIMENTIN. Moreover, Western blot showed that CXCL12 induced β-CATENIN and reduced E-CADHERIN in CAOV3 and IGROV1 cells, with R54 reverting these effects (Fig 3C and S3B Fig); of note in highly mesenchymal OVCAR8 cells, β-CATENIN was not induced by CXCL12 but still reduced by R54 (Fig 3C) while E-CADHERIN was undetectable (not shown). CXCL12 was unable to induce EMT in IGROV1 CXCR4-KO (S4A and S4B Fig), confirming the specific role of CXCR4 to promote CXCL12 mediated EMT in ovarian cancer and the ability of R54 in hinder this process.

### 3.4 R54 inhibits CXCR4 signalling pathway

**Fig 4:**
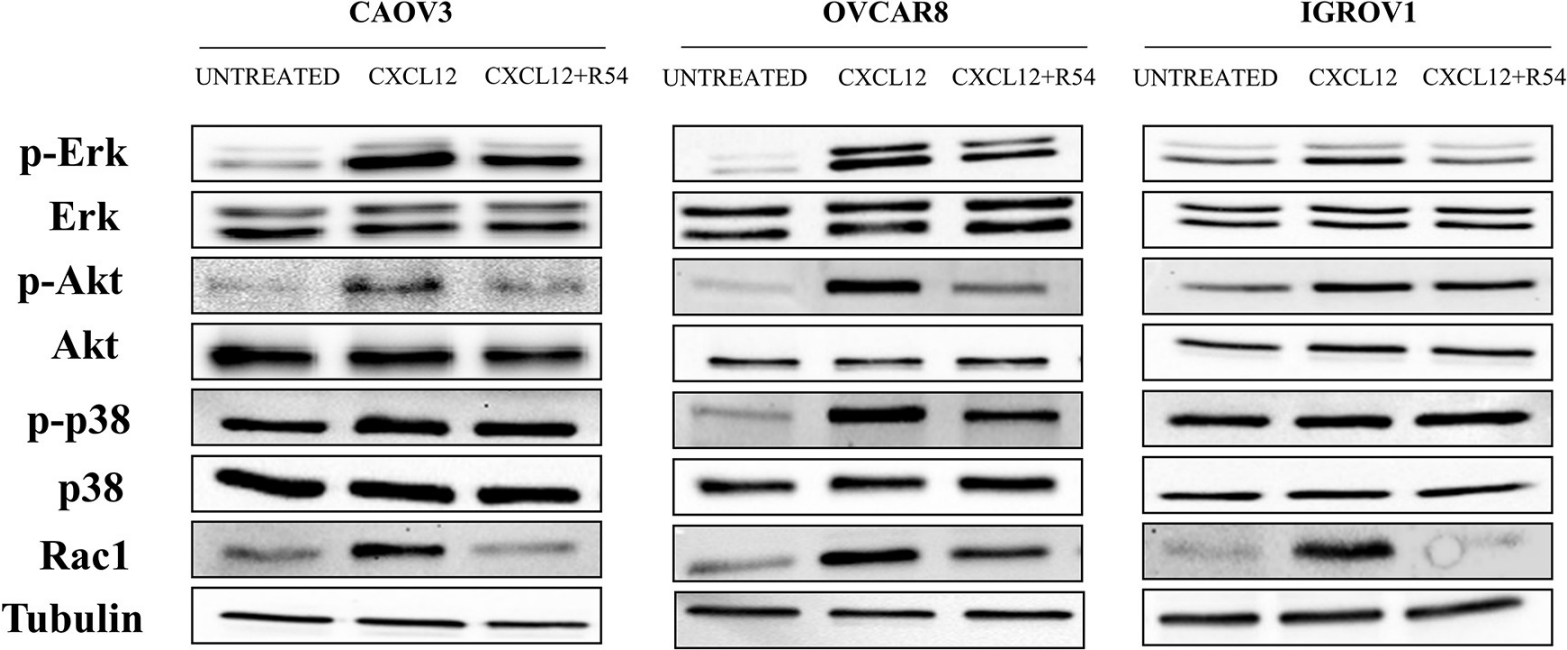
R54 inhibited EMT signaling in OC cell lines. Western blot for p-ERK, p-AKT, p-p38 and RAC1 protein levels after 10 minutes of stimulation with CXCL12 100 ng/mL ± R54 100 nM.

After binding with CXCL12, CXCR4 activates downstream signaling pathways ERK1/2, AKT, RHO/RAC1/CDC42 [8]. To define the activity of R54 on CXCR4 downstream signaling, the phosphorylation of ERK1/2, AKT and p38 was evaluated in CAOV3, OVCAR8 and IGROV1. As shown in Fig 4, CXCL12 induced p-ERK1/2 and p-AKT while R54 reverted it in CAOV3 and OVCAR8. In IGROV1, the inhibitory effect of R54 was observed only on CXCL12 induced ERK1/2 phosphorylation. p-p38 was induced by CXCL12 and reverted through R54 only in OVCAR8 (Fig 4). RAC1 inhibition repressed EMT in mesenchymal-like ovarian cancer cells [19]. CXCL12 induced RAC1, while R54 reverted the CXCL12-RAC induction, mainly in IGROV1 (Fig 4). In IGROV1 CXCR4-KO ERK, AKT, p38 and RAC1 were not phosphorylated or induced by CXCL12 (S4C Fig). Intriguingly, R54 does not totally inhibit CXCR4 downstream pathways probably due to the presence of CXCR7 receptor which also binds CXCL12 and activates proliferation and invasion signaling [20]; this effect is particularly evident in IGROV1 cells that express higher CXCR7 among assessed cell lines.

### 3.5 R54 impairs ovarian cancer cell growth and sensitizes ovarian cancer cells to CDPP and PTX

**Fig 5:**
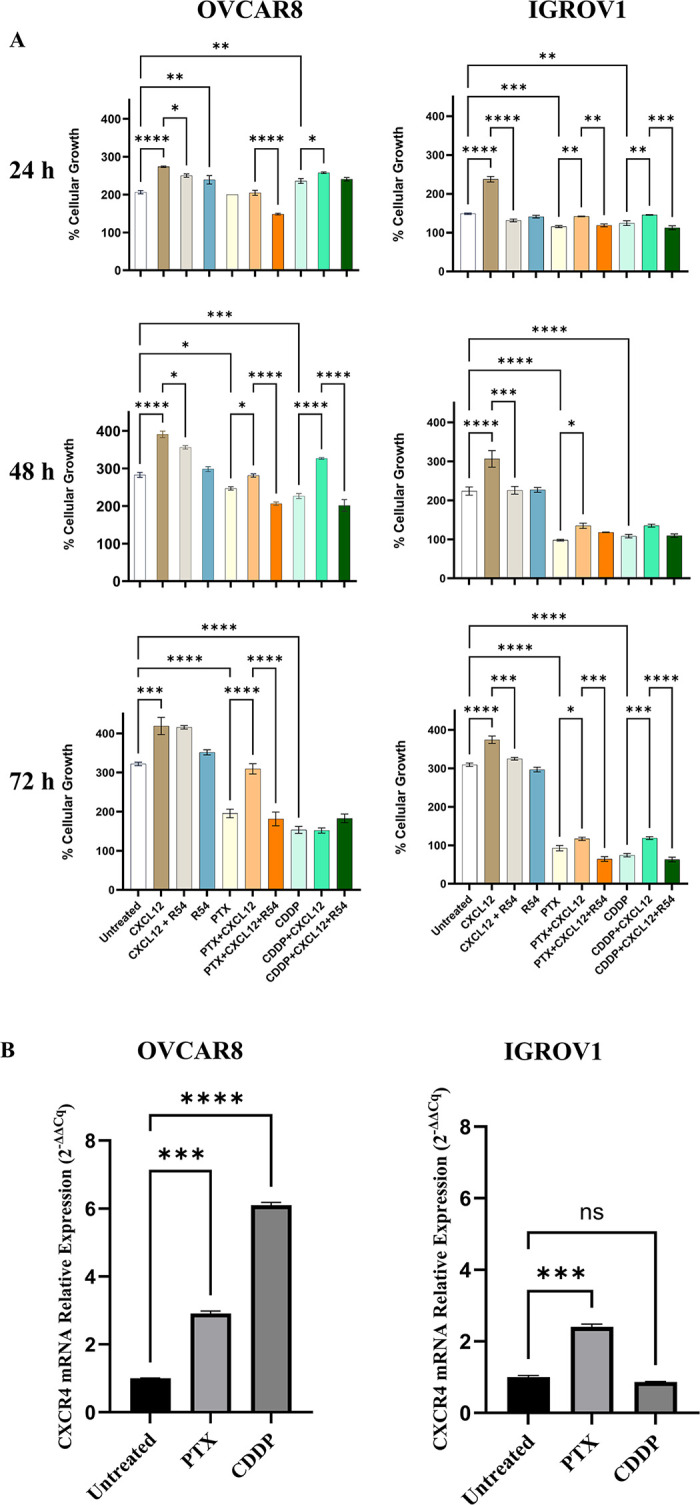
R54 counteracts CXCL12-mediated resistance to cisplatin and paclitaxel in ovarian cancer cells. (A) Growth curves of OVCAR8 and IGROV1 in the presence of CXCL12 (100ng/ml), R54 (100nM), Cisplatin 2 μM in OVCAR8 and 250 nM in IGROV1 and Paclitaxel 3 nM in OVCAR8 and 2 nM in IGROV1. Cells were counted after 24, 48 and 72 hours of culture. Data are presented as mean values ± Standard Deviation. * denote p < 0,05, ** denote p < 0,01,*** denote p < 0,001, **** denote p < 0,0001; (B) qRT-PCR for CXCR4 in OVCAR8 and IGROV1 after treatment with Cisplatin 2 μM in OVCAR8 and 250 nM in IGROV1 and Paclitaxel 3 nM in OVCAR8 and 2 nM in IGROV1. Gene expression was calculated using a 2^-ΔΔCq^ method (normalized to the mean of GUSB and α-TUBULIN in OVCAR8 and to the mean of GUSB and B2M in IGROV1) as the fold increase compared with untreated cells. Data are presented as mean values ± Standard Deviation. * denote p < 0,05, ** denote p < 0,01,*** denote p < 0,001, **** denote p < 0,0001.

First line therapy in OC patients relies on cisplatin (CDDP) and paclitaxel (PTX). CXCL12-induced mesenchymal transition confers resistance to CDDP and PTX [21]. To evaluate the impact of R54 in ovarian cancer cells sensitivity to chemotherapy, CAOV3, OVCAR8 and IGROV1 cells were treated with cisplatin (CDDP) and paclitaxel (PTX) and CXCL12 dependent growth was evaluated in the presence of R54 (Fig 5A and S6A Fig). As shown in Fig 5A, CXCL12 induced OVCAR8 and IGROV1 proliferation, and R54 impaired it. CXCL12 induced CAOV3 proliferation at 48 hours and R54 impaired it (S6A Fig). As expected, CXCL12 mediates resistance to CDDP and PTX in EOC cell lines increasing cancer cell proliferation in presence of chemotherapeutic agents. Targeting CXCR4 with R54 reverts CXCL12-induced cancer cell growth in presence of CDDP/PTX, particularly in OVCAR8 and IGROV1 treated with PTX potentiating the effect of chemotherapeutic agents (Fig 5A and S6A Fig). Since CXCL12 induces resistance to chemotherapeutics in the evaluated cells we hypothesize that chemotherapeutic agents may promote CXCR4 expression. Significant increase in CXCR4 expression was shown after treatment with PTX particularly in OVCAR8 and IGROV1 while Cisplatin induced a significant increase in CXCR4 only in OVCAR8 not modifying CXCR4 expression in IGROV1 and CAOV3 (Fig 5B and S6B Fig).

## 4. Discussion

In the present study, CXCR4 inhibition was evaluated in human ovarian cancer cells. CAOV3, OVCAR8 and IGROV1 expressed CXCR4, migrated toward CXCL12 and transduced the signal through p-ERK and p-AKT. The new peptide CXCR4 inhibitor, R54 [17] impaired CXCR4 activity reducing cell growth, migration and signalling in human ovarian cancer cells. Moreover, CXCL12 promoted epithelial to mesenchymal transition through VIMENTIN and BETA-CATENIN while R54 counteracted it. In addition, targeting CXCR4 improved chemosensitivity (CDDP-Paclitaxel), reverting CXCL12-induced resistance to chemotherapeutics. The metastatic potential of CXCR4 strongly correlates to the acquisition of a mesenchymal phenotype that confer migration capability to neoplastic cells [8]. It was previously demonstrated that CXCR4 expression favored EMT and accelerated tumor growth *in vivo* with CXCR4 inhibition affecting the EMT markers [22]. Previous studies demonstrated a correlation between RAC1 and CXCR4 in cell motility processes [23]. As RAC1 belongs to the RHO family of small guanosines triphosphatase (GTPase), its primary function is the regulation of actin cytoskeletal organization [24]. It regulates E-CADHERIN-mediated cell-cell adhesion [25] and induces EMT through ERK/MEK [19]. Acquisition of mesenchymal traits could compromise therapeutic response to chemo- or targeted therapies [26]. As R54 impairs mesenchymal transition CXCL12 induced, the study suggests that R54 may be added to chemotherapy treatment of ovarian cancer to reduce mesenchymal features such as migration/ invasiveness and increase chemotherapy sensitivity. Moreover, since immune desert tumors are enriched in EMT gene-set signatures [27] and considering CXCR4/CXCL12 ability in shaping TME toward immunosuppression [28–32], CXCR4 blockade may potentiate PD-1/PD-L1 immunotherapies by a dual action: reverting cancer cells mesenchymal phenotype on one hand and driving TME polarization toward immune-activation on the other hand.

## Supporting information

S1 Fig Expression of CXCL12 and CXCR4 in CAOV3, OVCAR8 and IGROV1. **(A)** qRT-PCR was performed to evaluate *CXCL12* expression in OC cell lines. Gene expression was calculated using 2^-ΔΔCq^ method (normalized to *GUSB*); **(B)** Immunofluorescence for CXCR4 was performed in CAOV3, OVCAR8 and IGROV1 cell lines.
(TIF)

S2 Fig IGROV1-CXCR4 gene knockout cells modestly migrated toward CXCL12. **(A)** qRT-PCR for *CXCR4* mRNA expression in sixteen clones of IGROV1 CXCR4-KO. Gene expression was calculated using 2^-ΔΔCq^ method (normalized to *α-TUBULIN)*. Data are presented as mean values ± Standard Deviation. *denote p<0,05, ** denote p<0,005, *** denote p<0,0005; **(B)** Flow cytometry CXCR4 in IGROV1 wild type versus IGROV1 CXCR4-KO; **(C**) Migration assay was performed in IGROV1 CXCR4-KO allowed to migrate for 16 hours toward 100 ng/ml CXCL12 +/- R54 100 nM. **** denote p < 0,0001.
(TIF)

S3 Fig R54 inhibited epithelial-mesenchymal transition. **(A)** qRT-PCR for EMT markers in human ovarian cancer cells were performed after stimulation with CXCL11 (100 ng/mL ± R54 100 nM). Gene expression was calculated using a 2^-ΔΔCq^ method (normalized to the mean of GUSB and α-TUBULIN in OVCAR8 and to the mean of GUSB and B2M in IGROV1 and CAOV3) as the fold increase compared with untreated cells. Data are presented as mean values ± Standard Deviation. * denote p < 0,05, ** denote p < 0,01,*** denote p < 0,001, **** denote p < 0,0001; **(B)** Western Blots for E- CADHERIN after 24 hours of stimulation with CXCL12 100 ng/mL ± R54 100 nM in CAOV3 and IGROV1 cells.
(TIF)

S4 Fig CXCR4 gene knockout impaired EMT-CXCL12 induced in IGROV1 CXCR4-KO cell line. **(A)** qRT-PCR to evaluate *VIMENTIN* and *SNAIL1* mRNA expression in IGROV1 CXCR4-KO exposed to 100 ng/mL CXCL12 in presence and absence of 100 nM R54. Gene expression was calculated using 2^-ΔΔCq^ method (normalized to the mean of *GUSB* and *B2M*). Data are presented as mean values ± standard deviation. *denote p<0,05; **(B)** Western blotting to evaluate the expression of BETA-CATENIN and VIMENTIN in presence of 100 nM R54 after stimulation with 100 ng/mL CXCL12 in IGROV1 CXCR4-KO. **(C)** Western blotting analysis of p-ERK, p-AKT, p-p38 and RAC1 in absence or presence of 100 nM R54 after stimulation with 100 ng/mL CXCL12 in IGROV1 CXCR4-KO.
(TIF)

S5 Fig Single channel view of Fig 3B. Immunofluorescence for E-CADHERIN and VIMENTIN after treatment with CXCL12 +/- R54 in CAOV3, OVCAR8 and IGROV1 cells.
(TIF)

S6 Fig R54 counteracts proliferation and paclitaxel resistance CXCL12- mediated in CAOV3. **(A)** Growth curves of CAOV3 in the presence of CXCL12 (100ng/ml), R54 (100nM), Cisplatin 5 μM and Paclitaxel 1 nM. Cells were counted after 24, 48 and 72 hours of culture. Data are presented as mean values ± Standard Deviation. * denote p < 0,05, ** denote p < 0,01, *** denote p < 0,001, **** denote p < 0,0001; **(B)** qRT-PCR for CXCR4 in CAOV3 were performed after treatment with Cisplatin 5 μM and Paclitaxel 1 nM. Gene expression was calculated using a 2^-ΔΔCq^ method (normalized to the mean of GUSB and B2M) as the fold increase compared with untreated cells. Data are presented as mean values ± Standard Deviation. * denote p < 0,05.
(TIF)

S1 Raw images (PDF)
