# Supplementary figures and images for: The CXCR4 antagonist R54 targets epithelial-mesenchymal transition (EMT) in human ovarian cancer cells
Source: PLoS One. 2024 Dec 19;19(12):e0314735. doi: 10.1371/journal.pone.0314735 (PMC11658595; doi:10.1371/journal.pone.0314735)

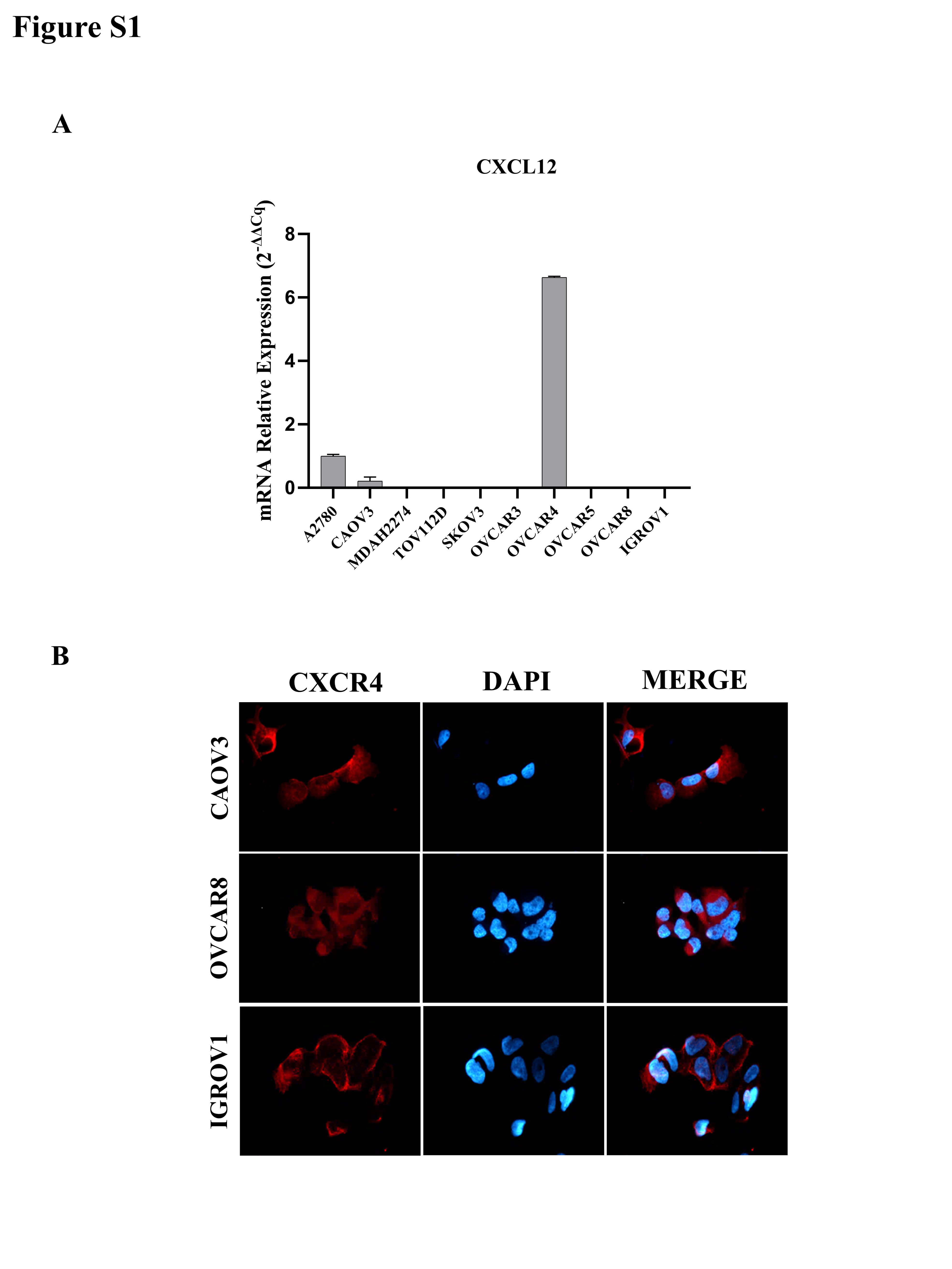

Supplement: S1 Fig — (A) qRT-PCR was performed to evaluate CXCL12 expression in OC cell lines. Gene expression was calculated using 2-ΔΔCq method (normalized to GUSB); (B) Immunofluorescence for CXCR4 was performed in CAOV3, OVCAR8 and IGROV1 cell lines. (TIF) [file pone.0314735.s001.tif]

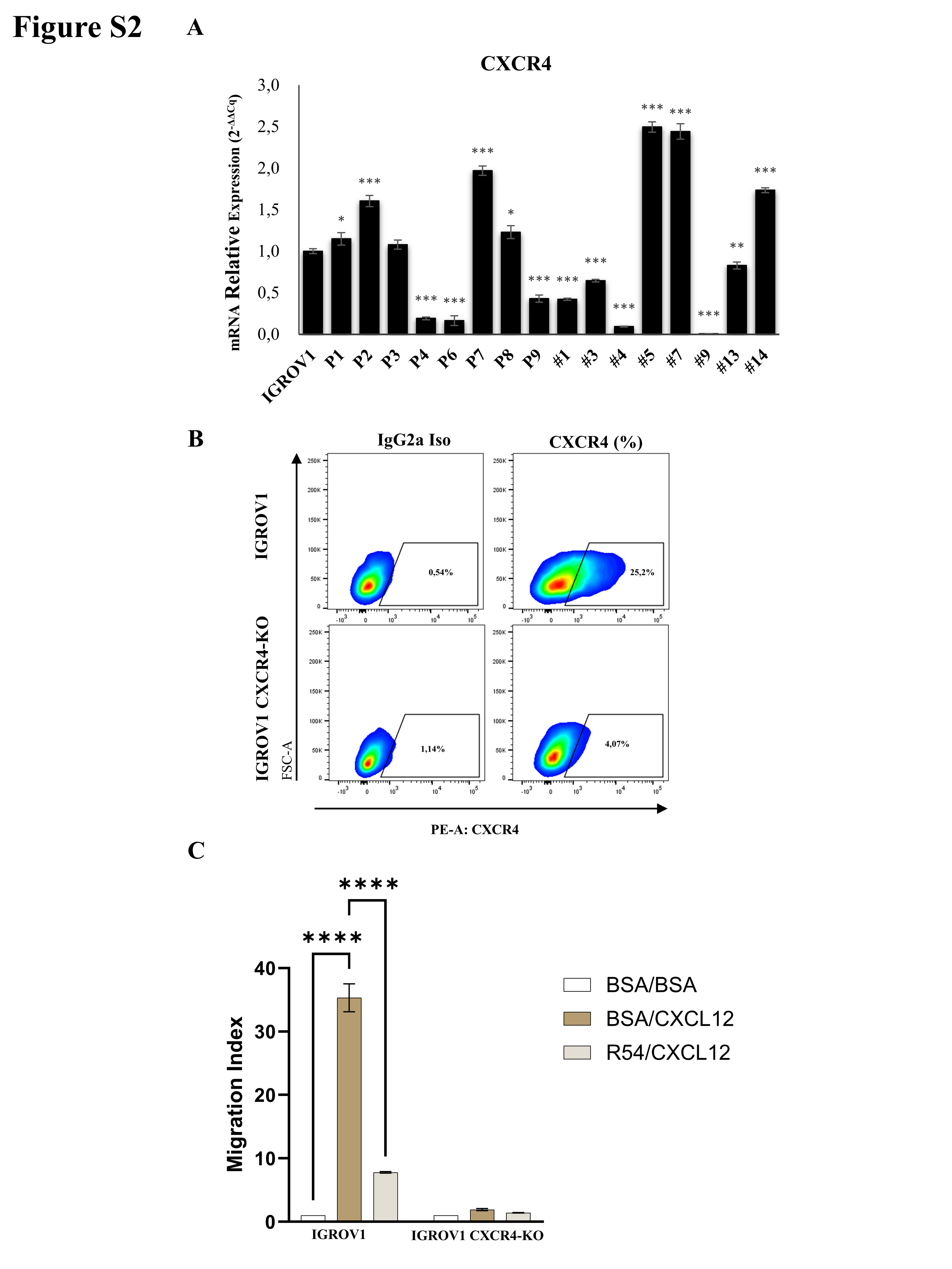

Supplement: S2 Fig — (A) qRT-PCR for CXCR4 mRNA expression in sixteen clones of IGROV1 CXCR4-KO. Gene expression was calculated using 2-ΔΔCq method (normalized to α-TUBULIN). Data are presented as mean values ± Standard Deviation. *denote p<0,05, ** denote p<0,005, *** denote p<0,0005; (B) Flow cytometry CXCR4 in IGROV1 wild type versus IGROV1 CXCR4-KO; (C) Migration assay was performed in IGROV1 CXCR4-KO allowed to migrate for 16 hours toward 100 ng/ml CXCL12 +/- R54 100 nM. **** denote p < 0,0001. (TIF) [file pone.0314735.s002.tif]

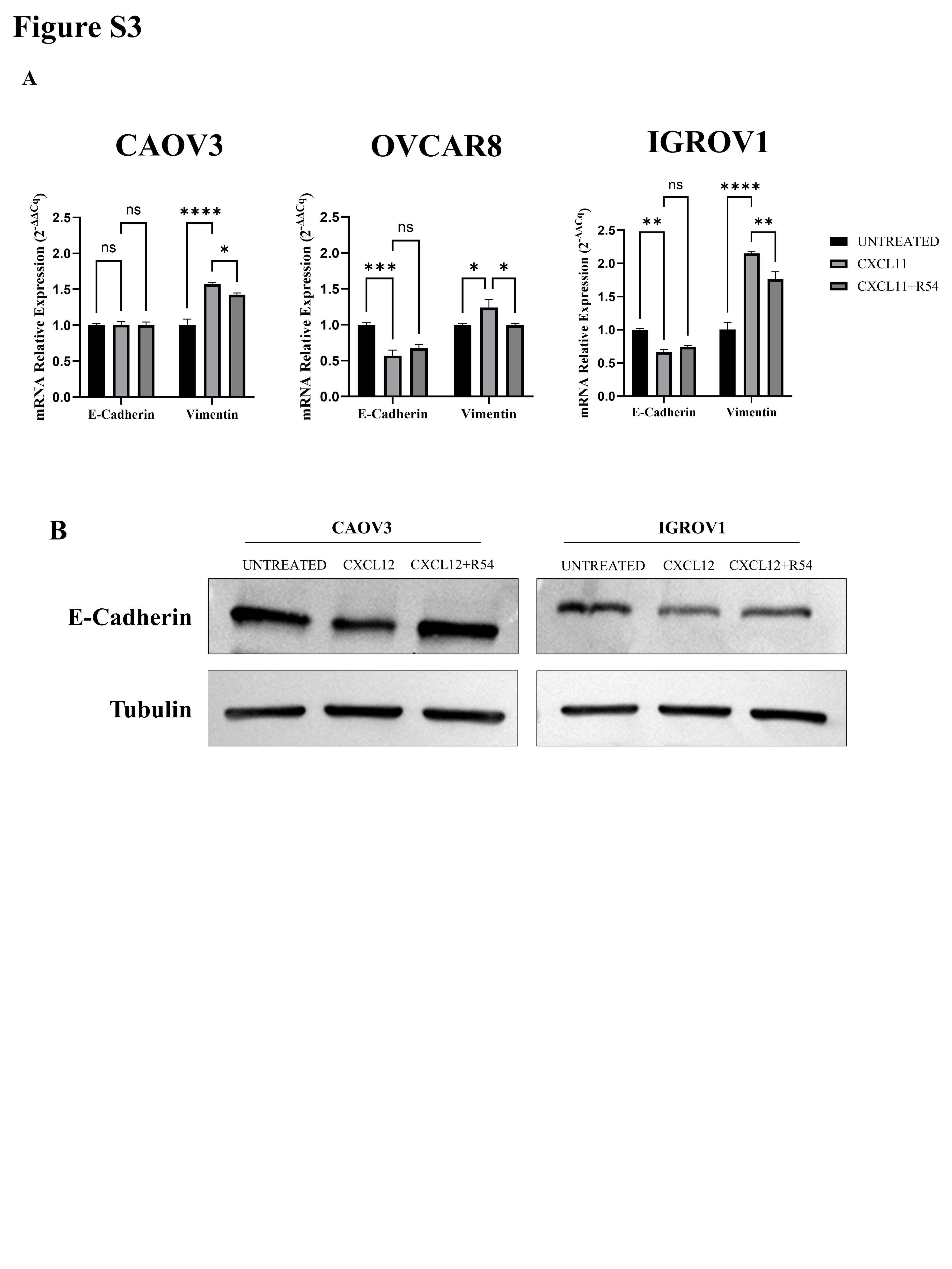

Supplement: S3 Fig — (A) qRT-PCR for EMT markers in human ovarian cancer cells were performed after stimulation with CXCL11 (100 ng/mL ± R54 100 nM). Gene expression was calculated using a 2-ΔΔCq method (normalized to the mean of GUSB and α-TUBULIN in OVCAR8 and to the mean of GUSB and B2M in IGROV1 and CAOV3) as the fold increase compared with untreated cells. Data are presented as mean values ± Standard Deviation. * denote p < 0,05, ** denote p < 0,01,*** denote p < 0,001, **** denote p < 0,0001; (B) Western Blots for E- CADHERIN after 24 hours of stimulation with CXCL12 100 ng/mL ± R54 100 nM in CAOV3 and IGROV1 cells. (TIF) [file pone.0314735.s003.tif]

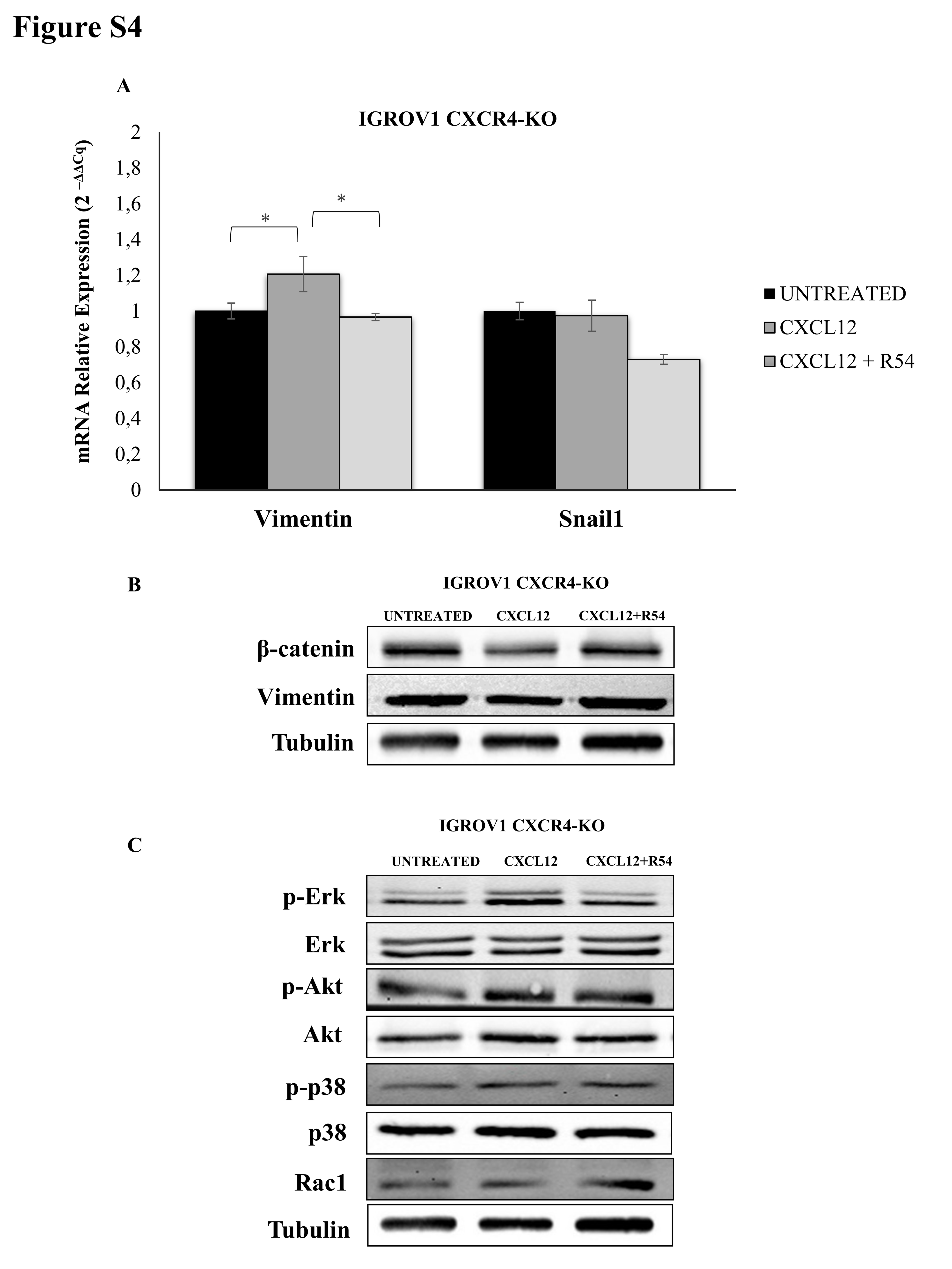

Supplement: S4 Fig — (A) qRT-PCR to evaluate VIMENTIN and SNAIL1 mRNA expression in IGROV1 CXCR4-KO exposed to 100 ng/mL CXCL12 in presence and absence of 100 nM R54. Gene expression was calculated using 2-ΔΔCq method (normalized to the mean of GUSB and B2M). Data are presented as mean values ± standard deviation. *denote p<0,05; (B) Western blotting to evaluate the expression of BETA-CATENIN and VIMENTIN in presence of 100 nM R54 after stimulation with 100 ng/mL CXCL12 in IGROV1 CXCR4-KO. (C) Western blotting analysis of p-ERK, p-AKT, p-p38 and RAC1 in absence or presence of 100 nM R54 after stimulation with 100 ng/mL CXCL12 in IGROV1 CXCR4-KO. (TIF) [file pone.0314735.s004.tif]

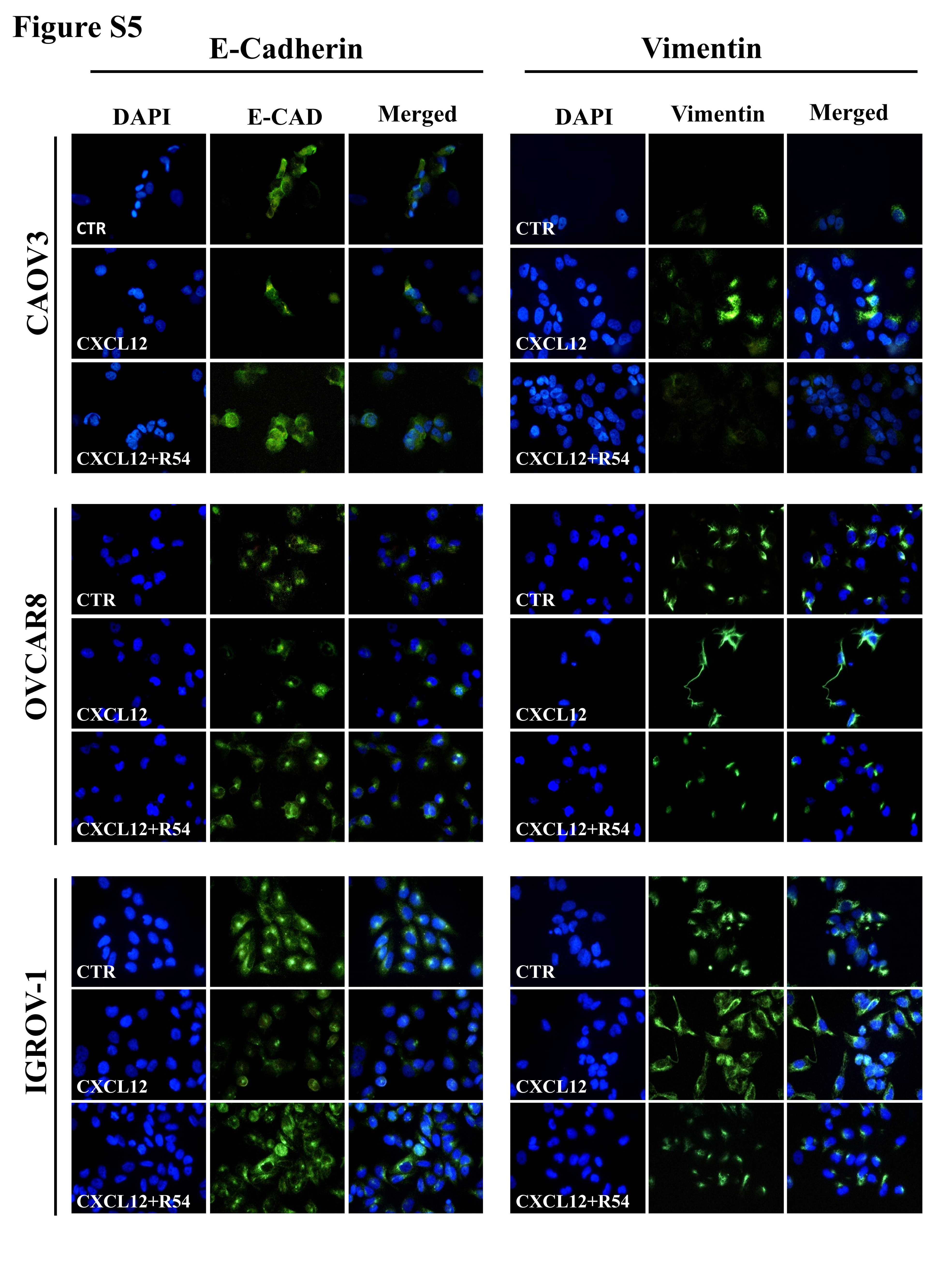

Supplement: S5 Fig — Immunofluorescence for E-CADHERIN and VIMENTIN after treatment with CXCL12 +/- R54 in CAOV3, OVCAR8 and IGROV1 cells. (TIF) [file pone.0314735.s005.tif]

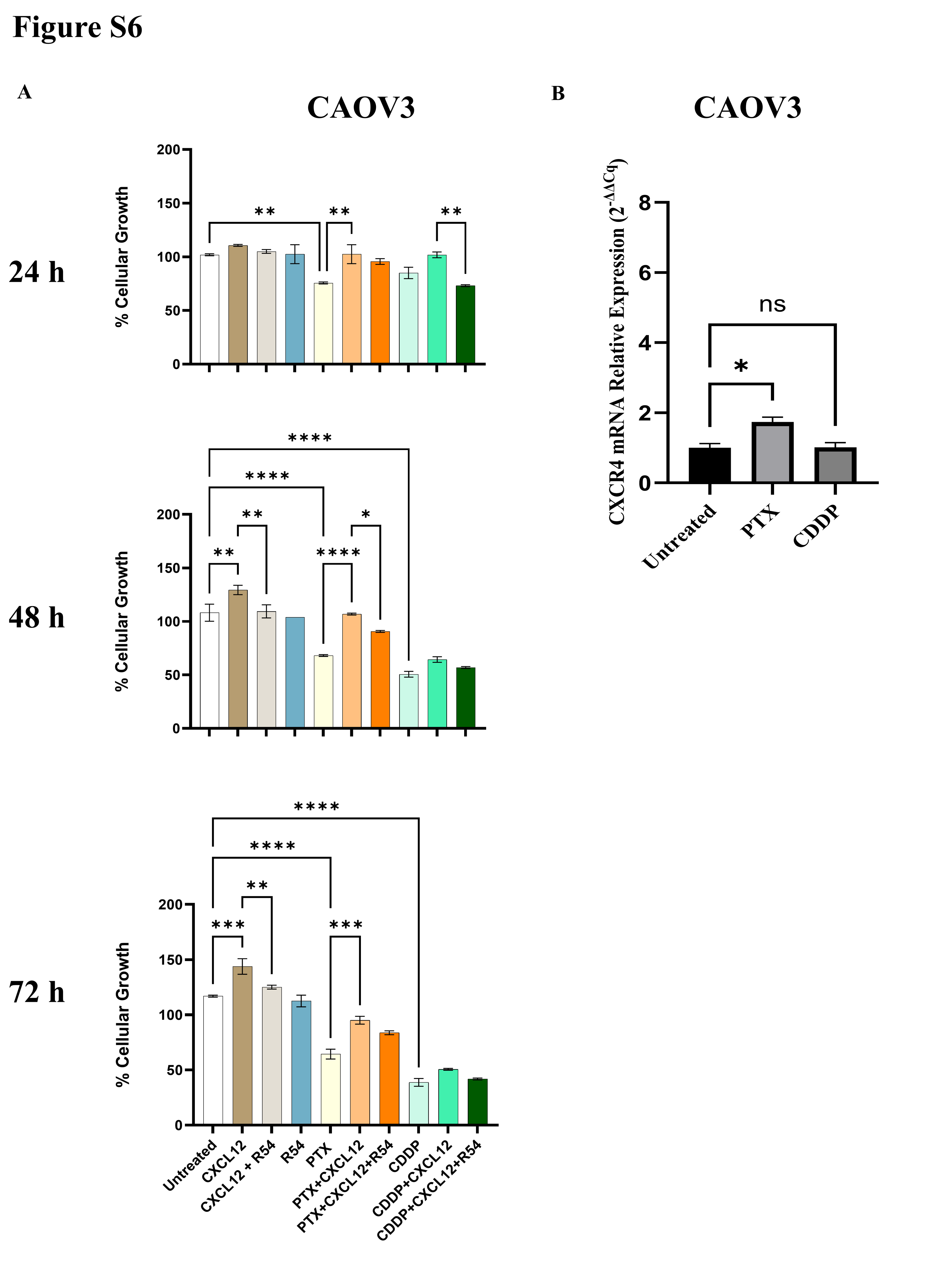

Supplement: S6 Fig — (A) Growth curves of CAOV3 in the presence of CXCL12 (100ng/ml), R54 (100nM), Cisplatin 5 μM and Paclitaxel 1 nM. Cells were counted after 24, 48 and 72 hours of culture. Data are presented as mean values ± Standard Deviation. * denote p < 0,05, ** denote p < 0,01, *** denote p < 0,001, **** denote p < 0,0001; (B) qRT-PCR for CXCR4 in CAOV3 were performed after treatment with Cisplatin 5 μM and Paclitaxel 1 nM. Gene expression was calculated using a 2-ΔΔCq method (normalized to the mean of GUSB and B2M) as the fold increase compared with untreated cells. Data are presented as mean values ± Standard Deviation. * denote p < 0,05. (TIF) [file pone.0314735.s006.tif]
