# Supplementary material for: The CXCR4 antagonist R54 targets epithelial-mesenchymal transition (EMT) in human ovarian cancer cells
Source: PLoS One. 2024 Dec 19;19(12):e0314735. doi: 10.1371/journal.pone.0314735 (PMC11658595; doi:10.1371/journal.pone.0314735)
Supplement: S1 Raw images — (PDF) [file pone.0314735.s007.pdf]

Figure 1B
CXCR4 in Ovarian cancer cell lines

Mw ladder
Lane 1: A2780
Lane 2: CAOV3
Lane 3: MDAH 2274
Lane 4: TOV112D
Lane 5: SKOV3
Lane 6: OVCAR3
Lane 7: OVCAR4
Lane 8: OVCAR5
Lane 9: OVCAR8
Lane 10: IGROV1
Lane 11: empty
Lane 12: CEM
Lane 13: JURKAT

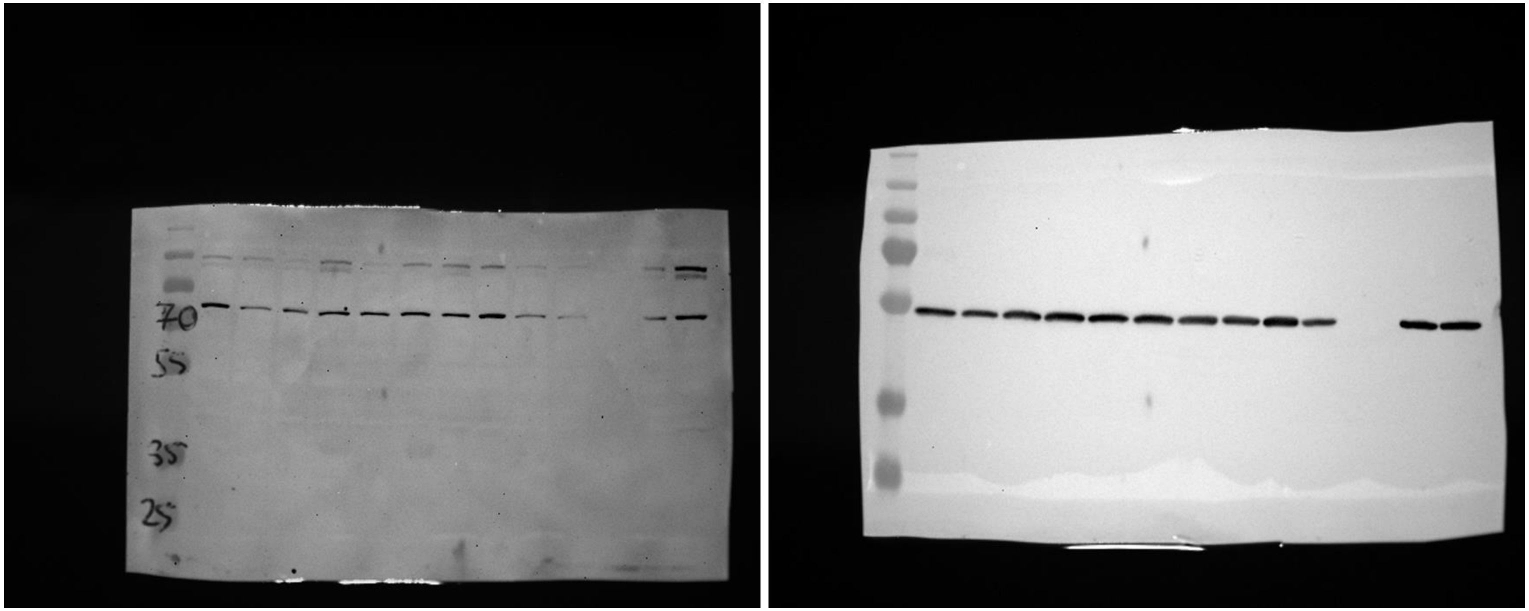

Images captured with iBright 1500 by Thermo Fisher Scientific

Figure 3C
beta-catenin CAOV3

Mw ladder
Lane 1: UNTREATED
Lane 2: CXCL12
Lane 3: CXCL12 + R54

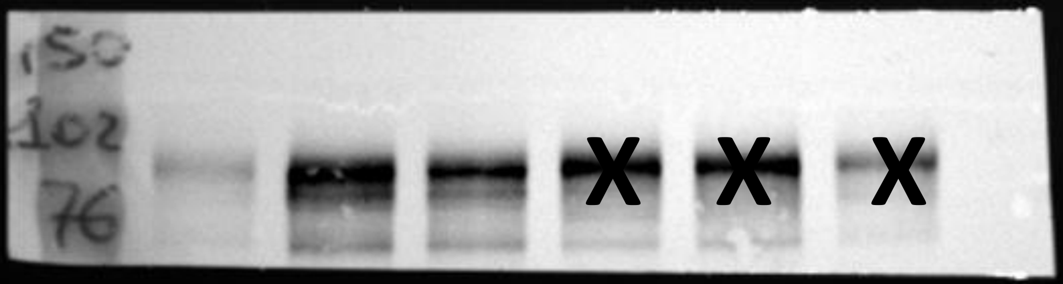

Images captured with iBright 1500 by Thermo Fisher Scientific

Figure 3C
beta-catenin OVCAR8

Mw ladder
Lane 4: UNTREATED
Lane 5: CXCL12
Lane 6: CXCL12 + R54

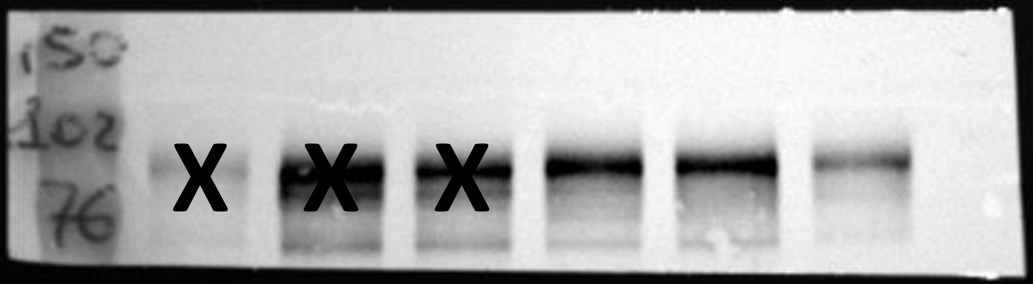

Images captured with iBright 1500 by Thermo Fisher Scientific

Figure 3C
beta-catenin IGROV1

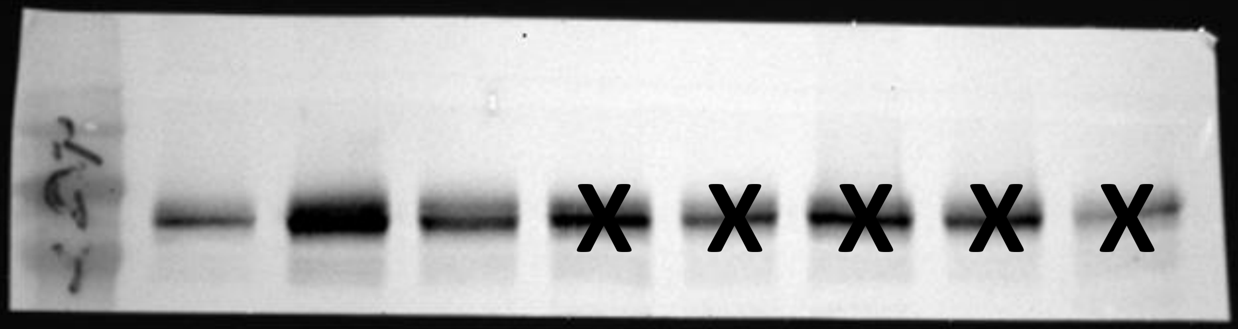

Mw ladder : R=Red 150 kDa, G=Green 102 KDa, Y=Yellow 76 KDa
-Lane 1: UNTREATED
-Lane 2: CXCL12
-Lane 3: CXCL12 + R54

Images captured with iBright 1500 by Thermo Fisher Scientific

Figure 3C
Vimentin CAOV3

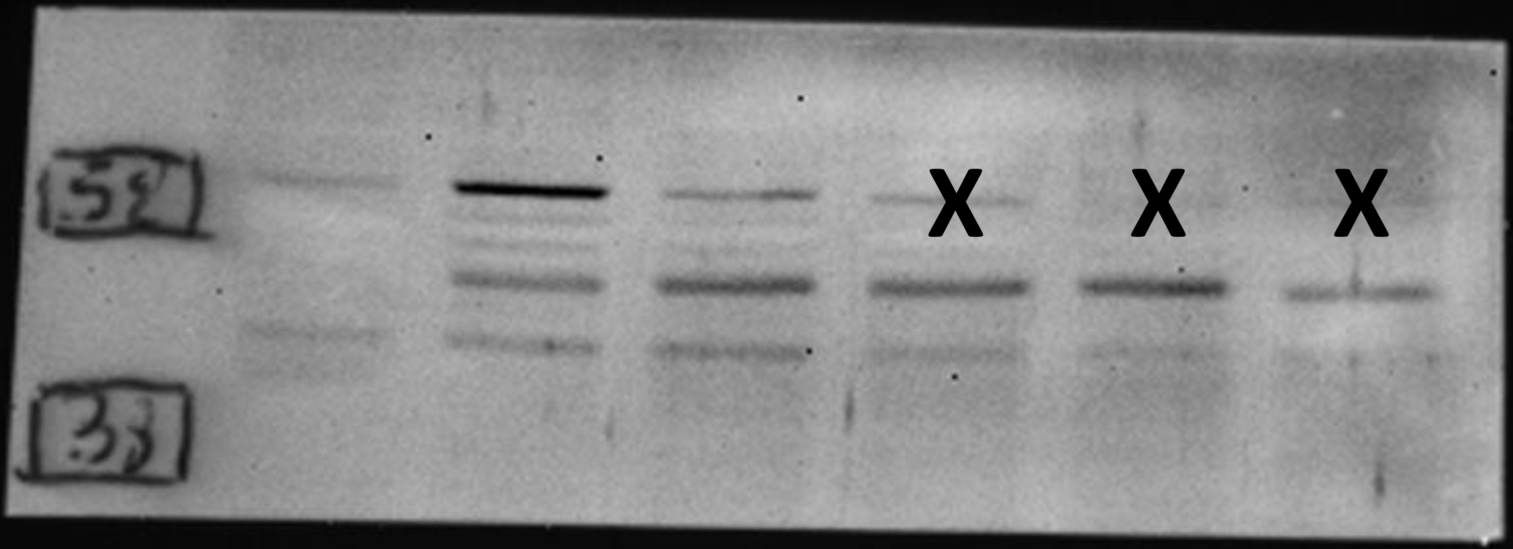

Mw ladder
Lane 1: UNTREATED
Lane 2: CXCL12
Lane 3: CXCL12 + R54

Images captured with iBright 1500 by Thermo Fisher Scientific

Figure 3C

Vimentin OVCAR8

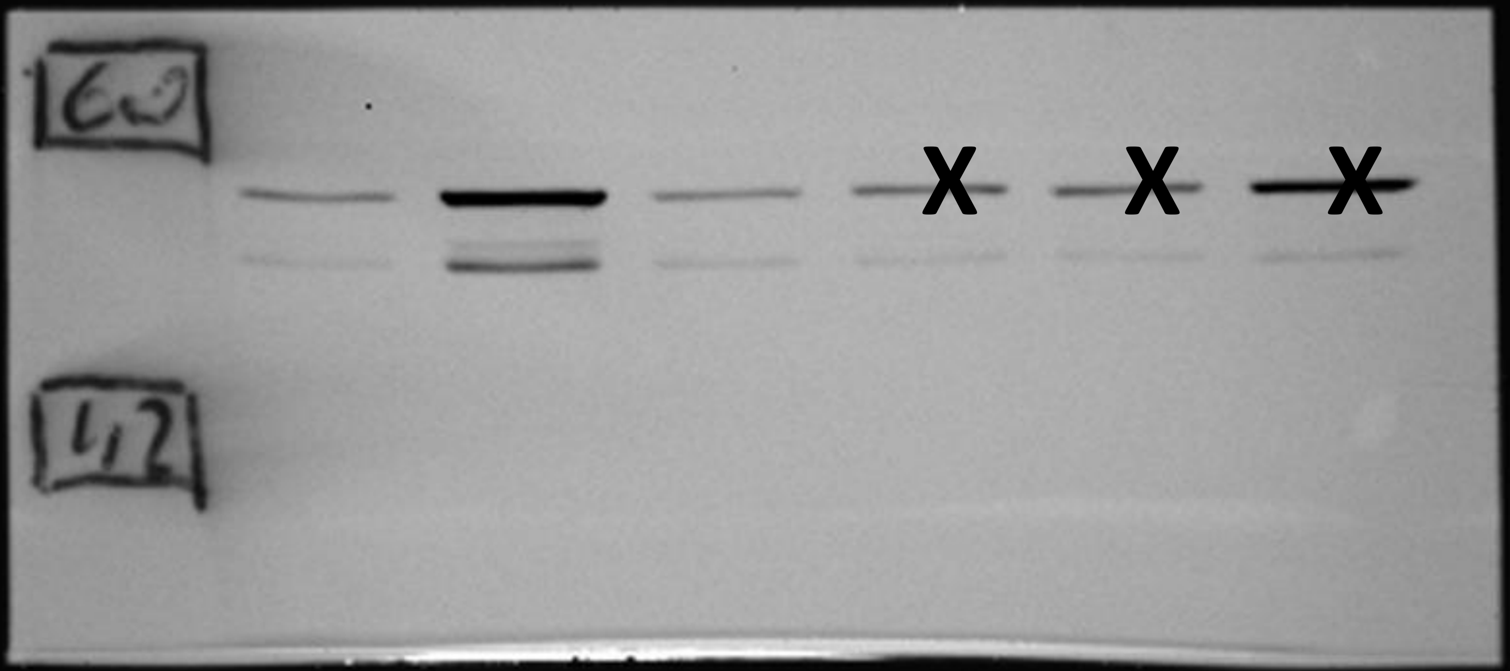

Mw ladder
Lane 1: UNTREATED
Lane 2: CXCL12
Lane 3: CXCL12 + R54

Images captured with iBright 1500 by Thermo Fisher Scientific

Figure 3C
Vimentin IGROV1

Mw ladder: P=purple 52 kDa, B=Blue 38 KDa
Lane 1: UNTREATED
Lane 2: CXCL12
Lane 3: CXCL12 + R54

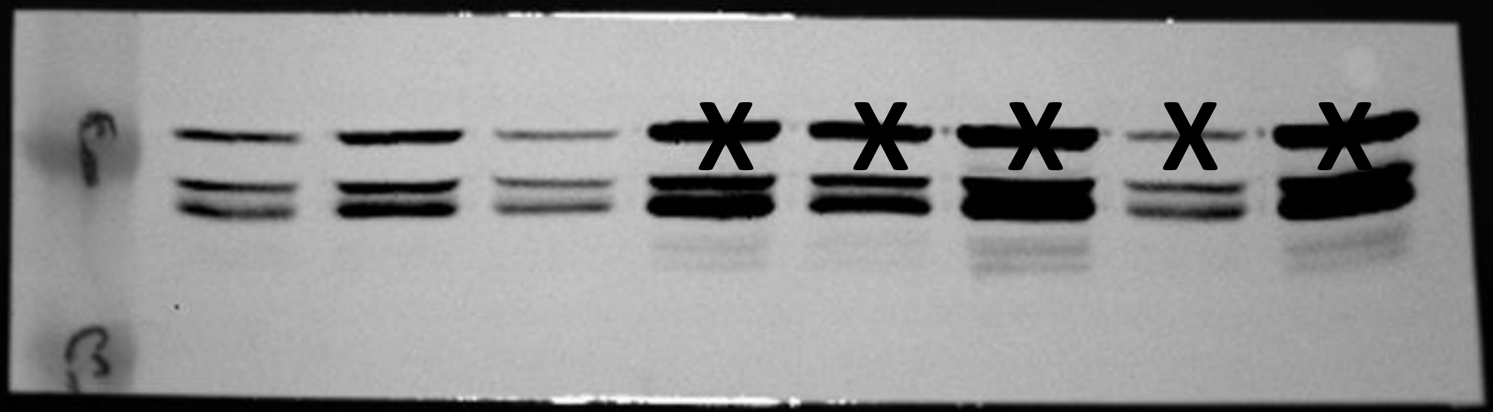

Images captured with iBright 1500 by Thermo Fisher Scientific

Figure 3C
Tubulin CAOV3

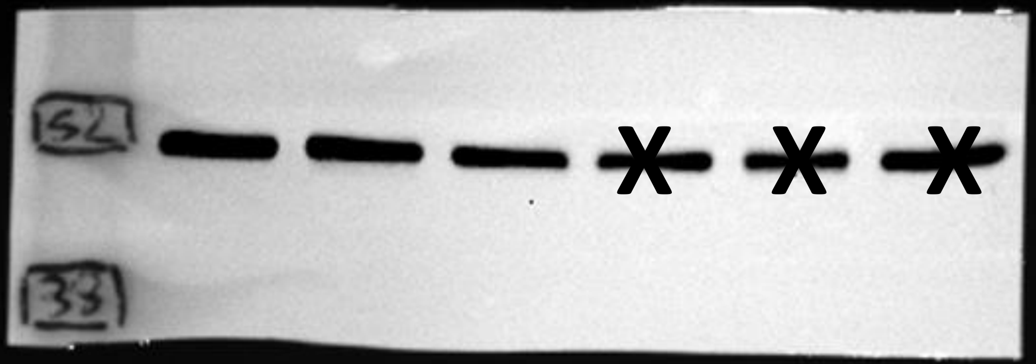

Mw ladder
Lane 1: UNTREATED
Lane 2: CXCL12
Lane 3: CXCL12 + R54

Images captured with iBright 1500 by Thermo Fisher Scientific

Figure 3C
Tubulin OVCAR8

Mw ladder
Lane 1: UNTREATED
Lane 2: CXCL12
Lane 3: CXCL12 + R54

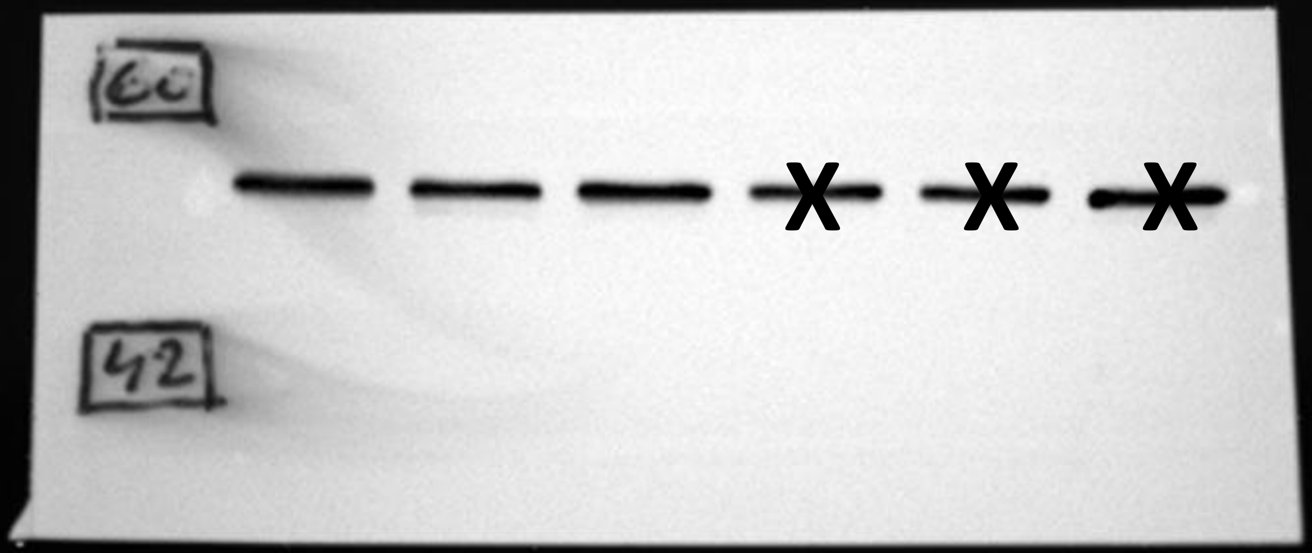

Images captured with iBright 1500 by Thermo Fisher Scientific

Figure 3C
Tubulin IGROV1

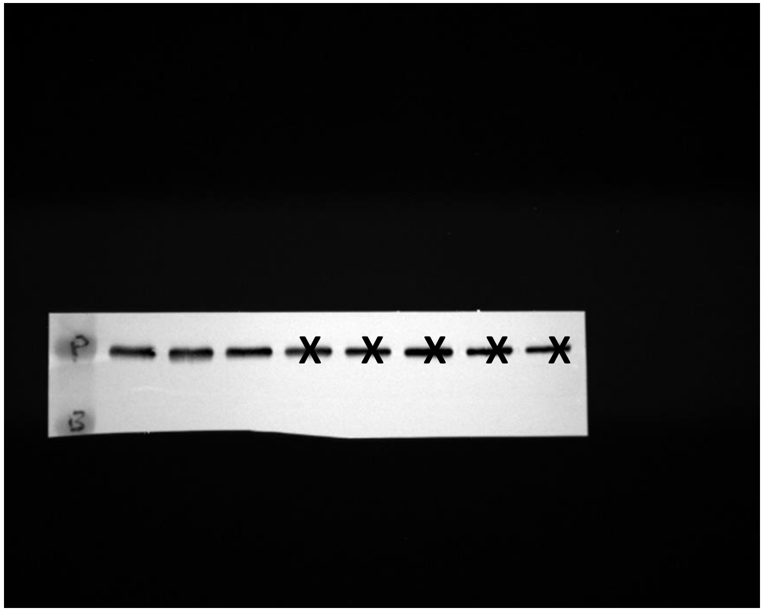

Mw ladder:P=purple 52 kDa, B=Blue 38 KDa
Lane 1: UNTREATED
Lane 2: CXCL12
Lane 3: CXCL12 + R54

Images captured with iBright 1500 by Thermo Fisher Scientific

Figure 4
p-Erk CAOV3

Mw ladder missing
Lane 1: UNTREATED
Lane 2: CXCL12
Lane 3: CXCL12 + R54

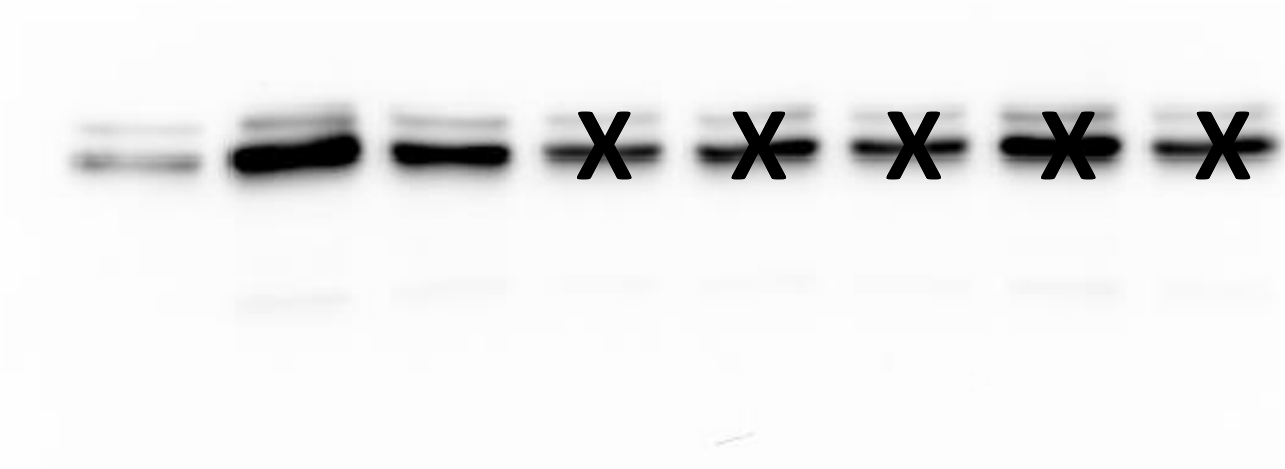

Images captured with iBright 1500 by Thermo Fisher Scientific

Figure 4
Erk tot CAOV3

Mw ladder missing
Lane 1: UNTREATED
Lane 2: CXCL12
Lane 3: CXCL12 + R54

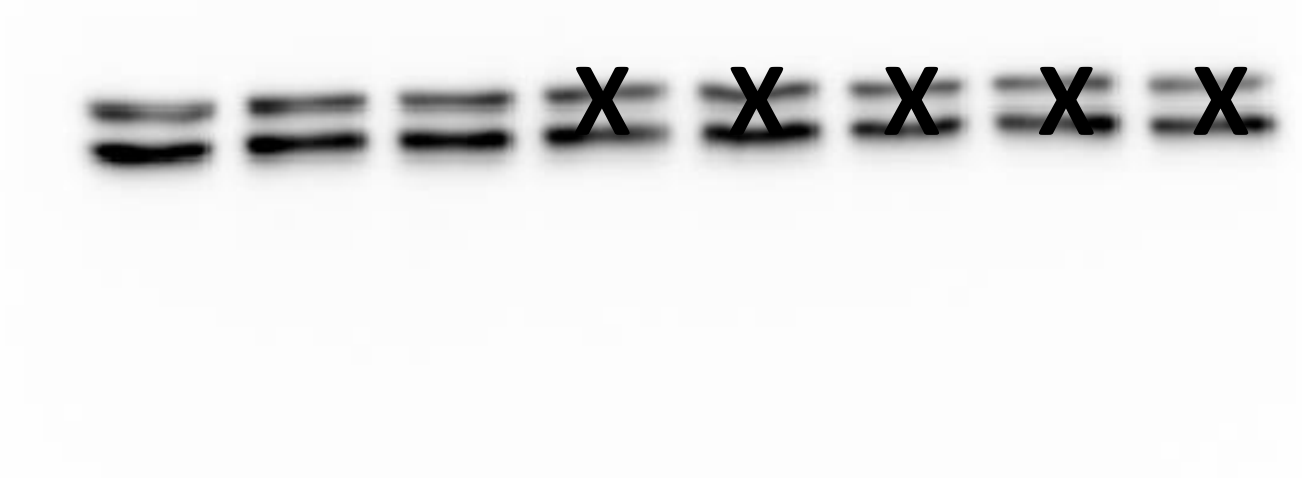

Images captured with iBright 1500 by Thermo Fisher Scientific

Figure 4
p-Akt CAOV3

Mw ladder missing
Lane 1: UNTREATED
Lane 2: CXCL12
Lane 3: CXCL12 + R54

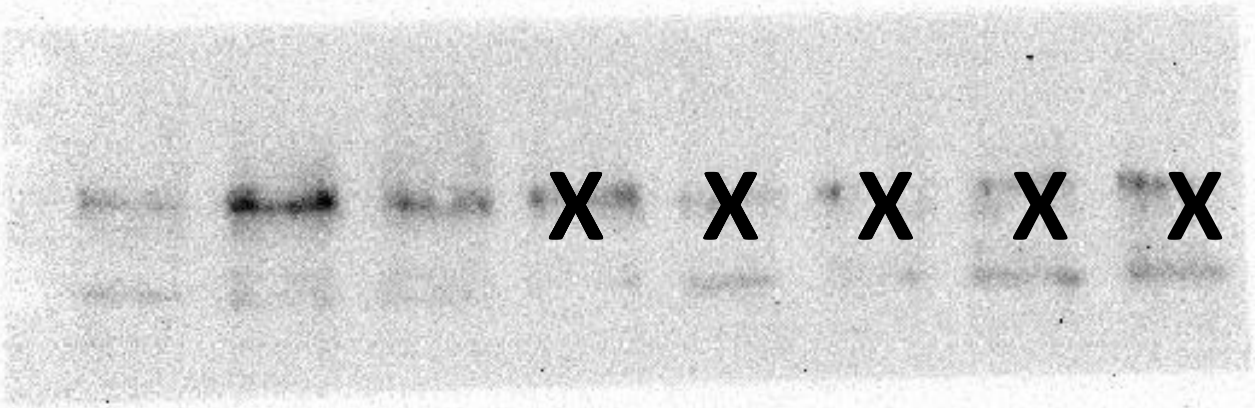

Images captured with iBright 1500 by Thermo Fisher Scientific

Figure 4
Akt tot CAOV3

Mw ladder missing
Lane 1: UNTREATED
Lane 2: CXCL12
Lane 3: CXCL12 + R54

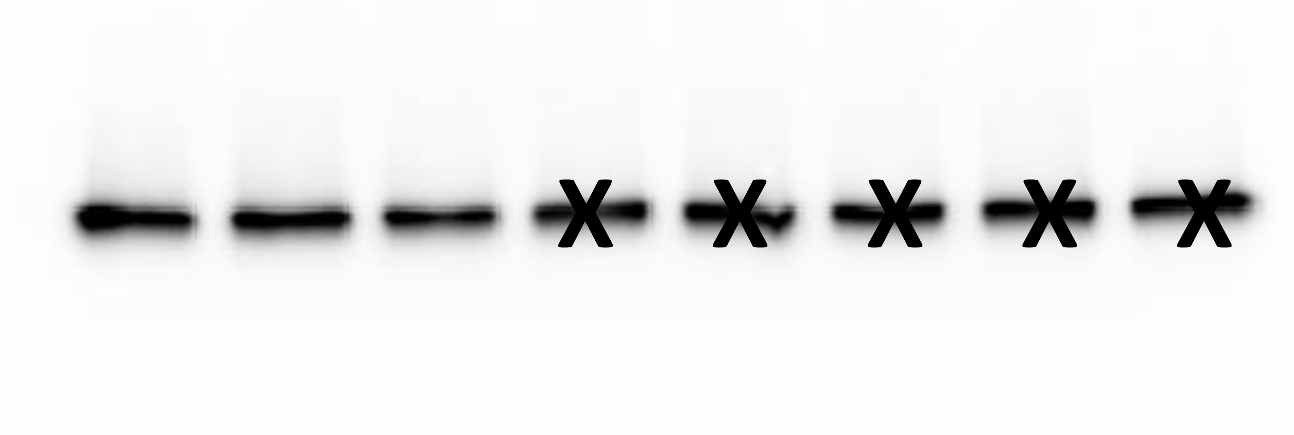

Images captured with iBright 1500 by Thermo Fisher Scientific

Figure 4
p-p38 CAOV3

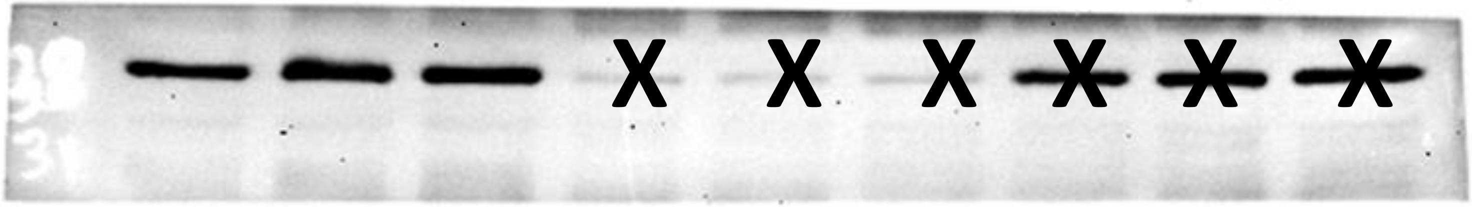

Mw ladder
Lane 1: UNTREATED
Lane 2: CXCL12
Lane 3: CXCL12 + R54

Images captured with iBright 1500 by Thermo Fisher Scientific

Figure 4
p38 tot CAOV3

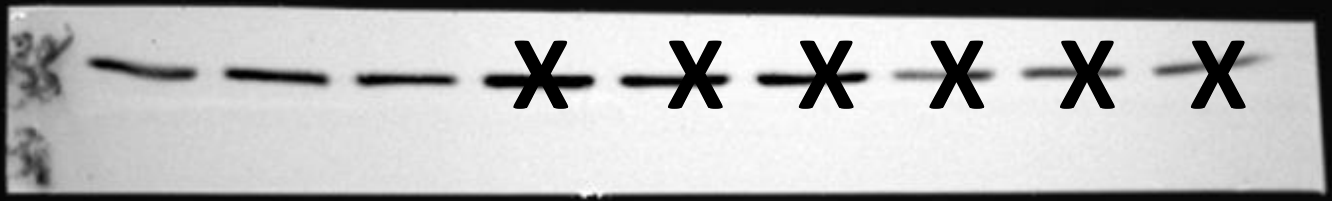

Mw ladder
Lane 1: UNTREATED
Lane 2: CXCL12
Lane 3: CXCL12 + R54

Images captured with iBright 1500 by Thermo Fisher Scientific

Figure 4
Rac1 CAOV3

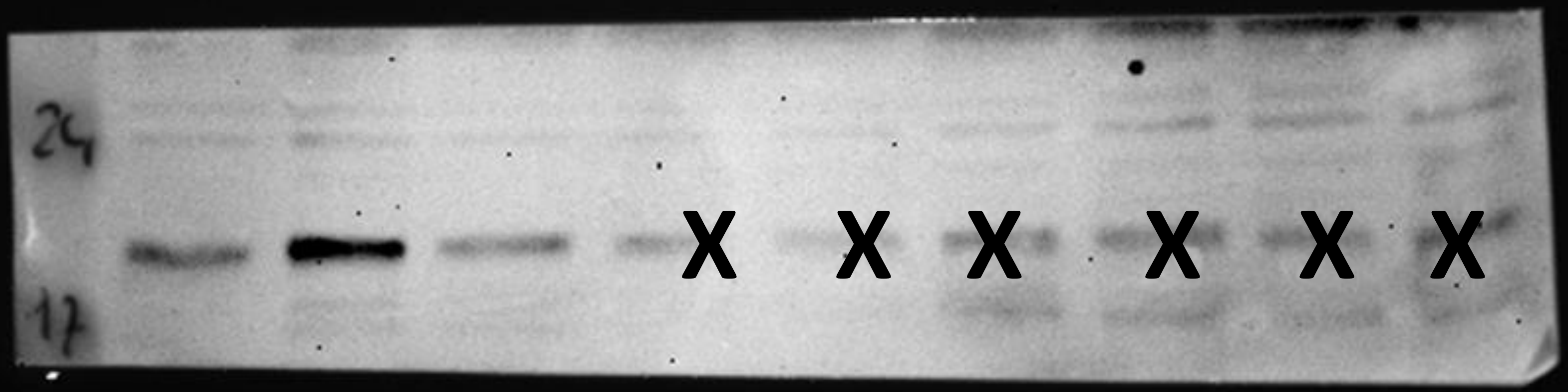

Mw ladder
Lane 1: UNTREATED
Lane 2: CXCL12
Lane 3: CXCL12 + R54

Images captured with iBright 1500 by Thermo Fisher Scientific

Figure 4
Tubulin CAOV3

Mw ladder
Lane 1: UNTREATED
Lane 2: CXCL12
Lane 3: CXCL12 + R54

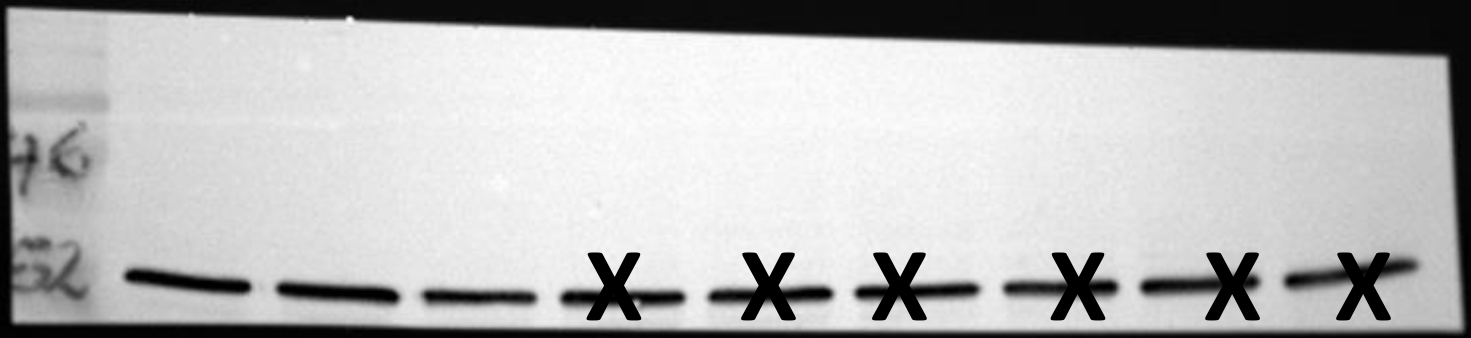

Images captured with iBright 1500 by Thermo Fisher Scientific

Figure 4
p-Erk OVCAR8

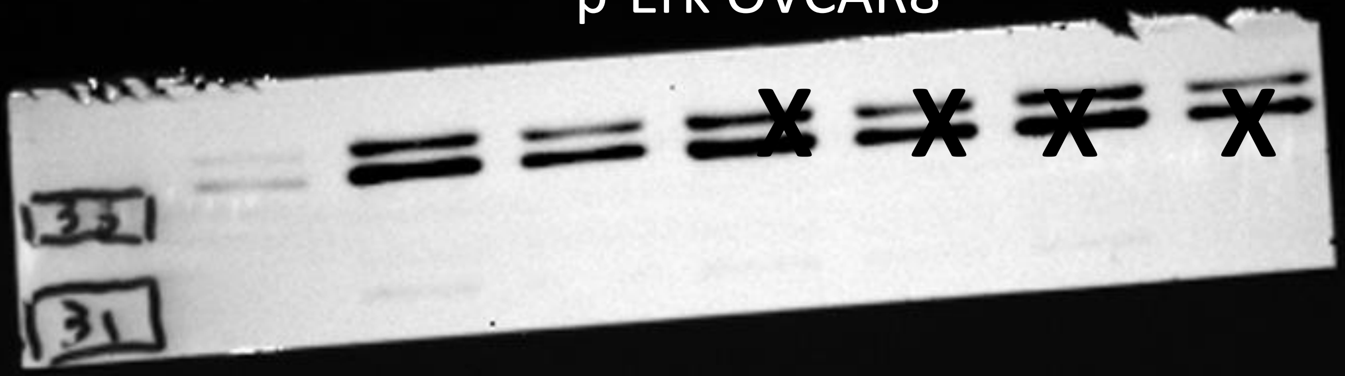

Mw ladder
Lane 1: UNTREATED
Lane 2: CXCL12
Lane 3: CXCL12 + R54

Images captured with iBright 1500 by Thermo Fisher Scientific

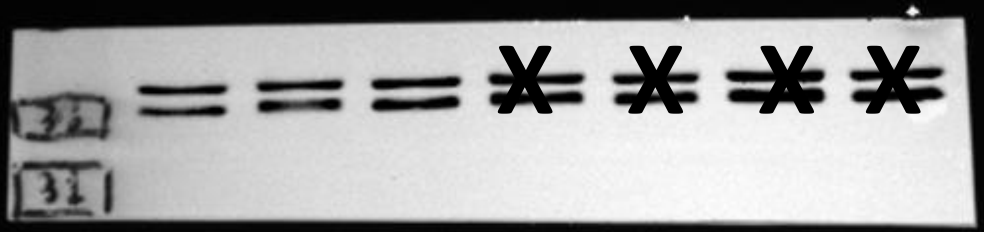

Figure 4
Erk tot OVCAR8

Mw ladder
Lane 1: UNTREATED
Lane 2: CXCL12
Lane 3: CXCL12 + R54

Images captured with iBright 1500 by Thermo Fisher Scientific

Figure 4
p-Akt OVCAR8

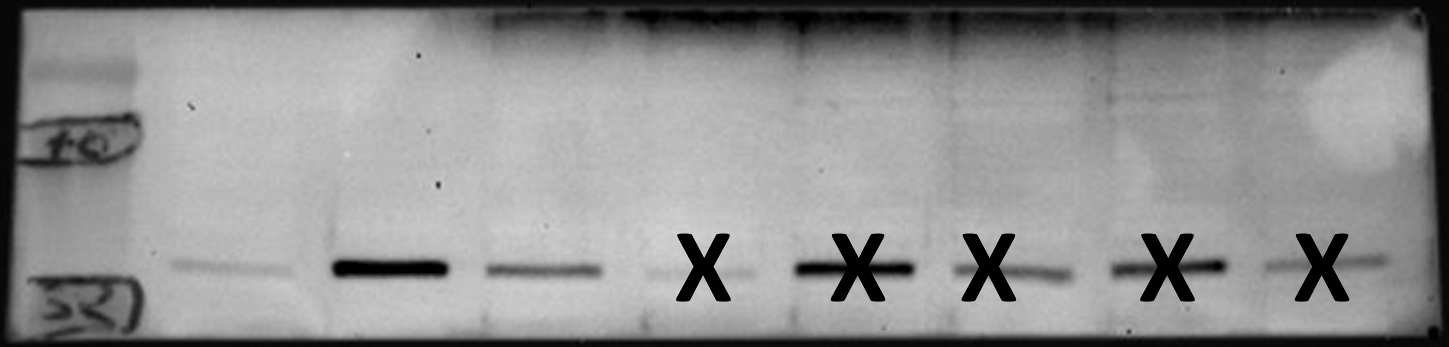

Mw ladder
Lane 1: UNTREATED
Lane 2: CXCL12
Lane 3: CXCL12 + R54

Images captured with iBright 1500 by Thermo Fisher Scientific

Figure 4
Akt tot OVCAR8

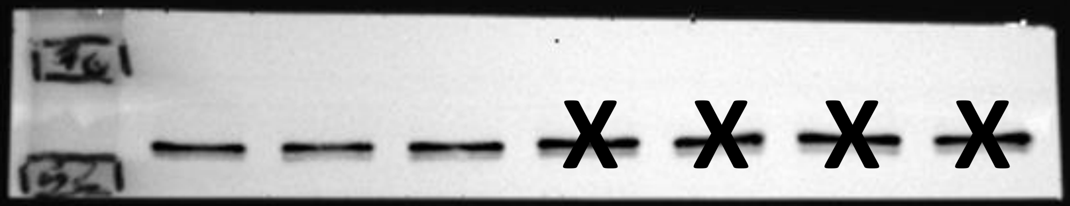

Mw ladder
Lane 1: UNTREATED
Lane 2: CXCL12
Lane 3: CXCL12 + R54

Images captured with iBright 1500 by Thermo Fisher Scientific

Figure 4
p-p38 OVCAR8

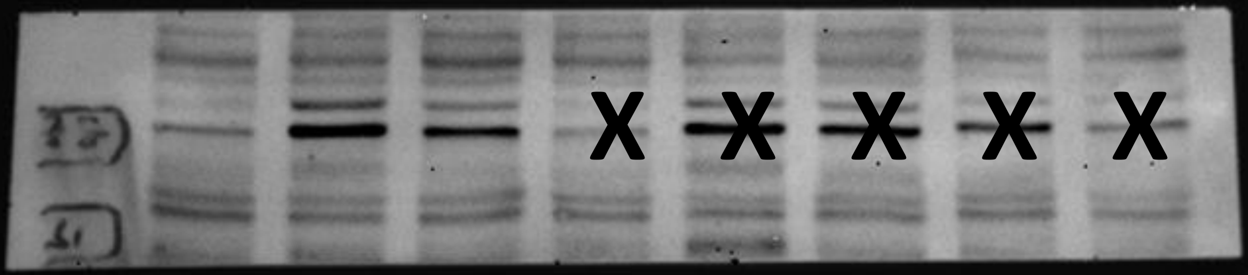

Mw ladder
Lane 1: UNTREATED
Lane 2: CXCL12
Lane 3: CXCL12 + R54

Images captured with iBright 1500 by Thermo Fisher Scientific

Figure 4
p38 tot OVCAR8

Mw ladder
Lane 1: UNTREATED
Lane 2: CXCL12
Lane 3: CXCL12 + R54

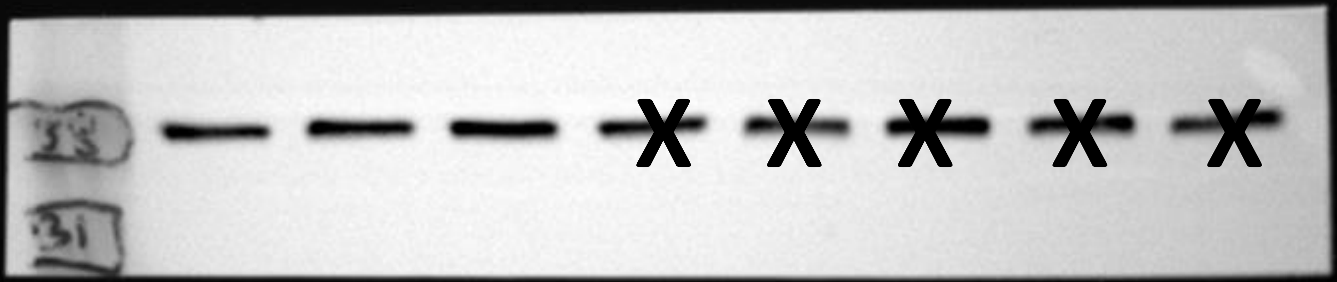

Images captured with iBright 1500 by Thermo Fisher Scientific

Figure 4
Rac1 OVCAR8

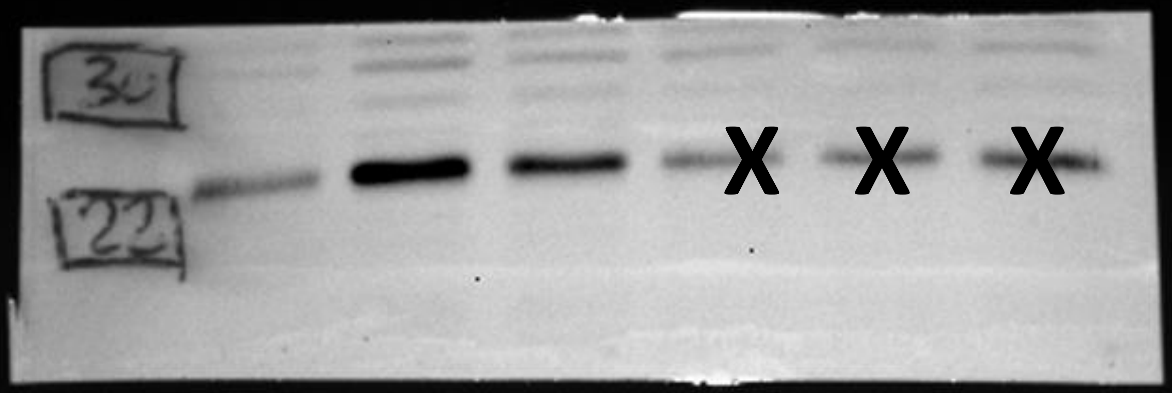

Mw ladder
Lane 1: UNTREATED
Lane 2: CXCL12
Lane 3: CXCL12 + R54

Images captured with iBright 1500 by Thermo Fisher Scientific

Figure 4
Tubulin OVCAR8

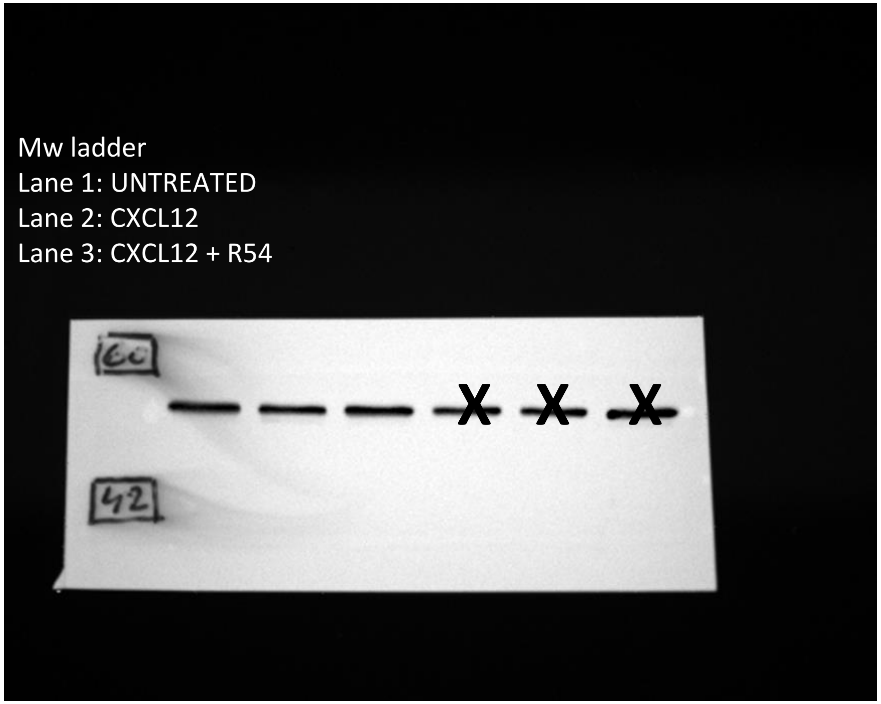

Images captured with iBright 1500 by Thermo Fisher Scientific

Figure 4

p-Erk IGROV1

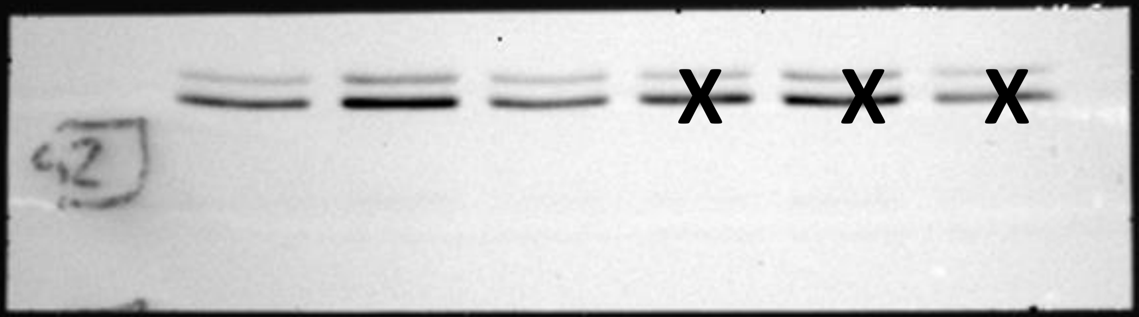

Mw ladder
Lane 1: UNTREATED
Lane 2: CXCL12
Lane 3: CXCL12 + R54

Images captured with iBright 1500 by Thermo Fisher Scientific

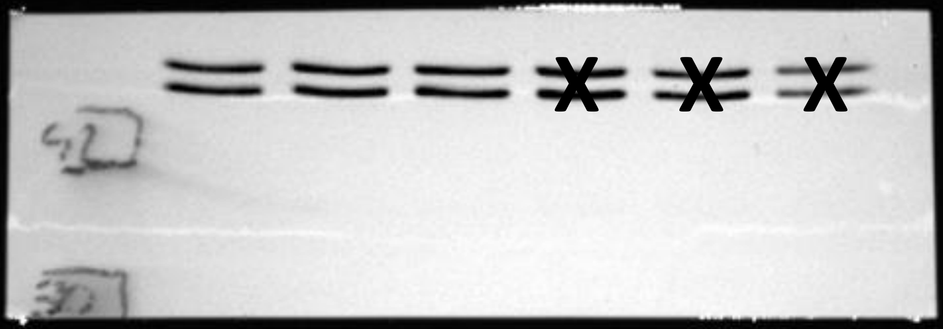

Figure 4
Erk tot IGROV1

Mw ladder
Lane 1: CTR
Lane 2: CXCL12
Lane 3: CXCL12 + R54

Images captured with iBright 1500 by Thermo Fisher Scientific

Figure 4
p-AKT IGROV1

Mw ladder
Lane 1: UNTREATED
Lane 2: CXCL12
Lane 3: CXCL12 + R54

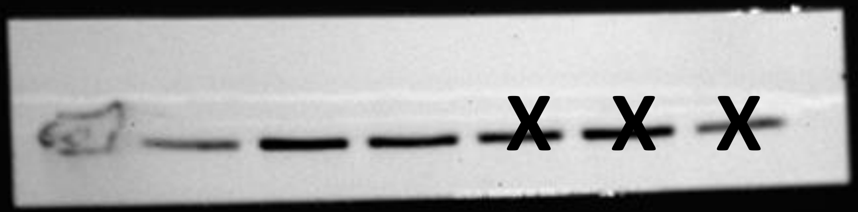

Images captured with iBright 1500 by Thermo Fisher Scientific

Fig 4
Akt tot IGROV1

Mw ladder
Lane 1: UNTREATED
Lane 2: CXCL12
Lane 3: CXCL12 + R54

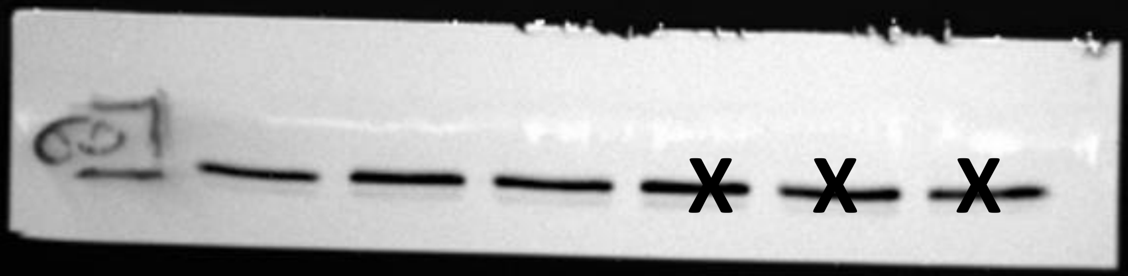

Images captured with iBright 1500 by Thermo Fisher Scientific

Figure 4
p-p38 IGROV1

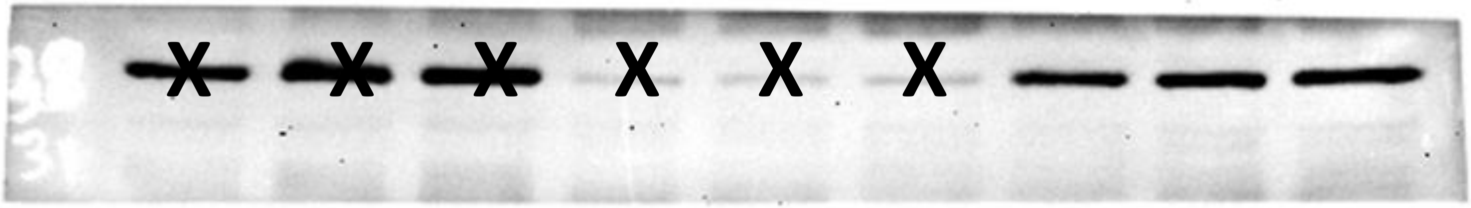

Mw ladder
Lane 7: UNTREATED
Lane 8: CXCL12
Lane 9: CXCL12 + R54

Images captured with iBright 1500 by Thermo Fisher Scientific

Figure 4
p38 tot IGROV1

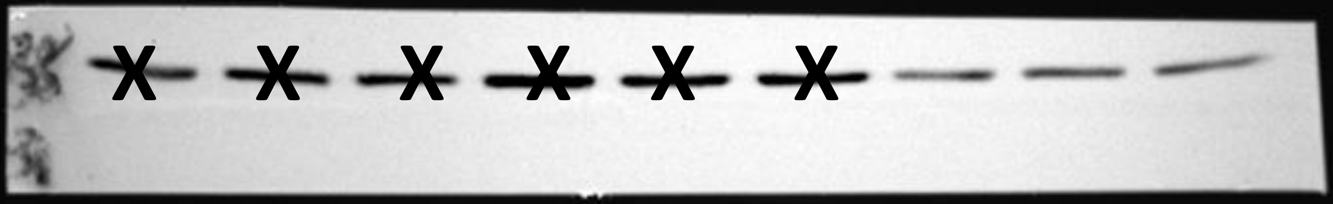

Mw ladder
Lane 7: UNTREATED
Lane 8: CXCL12
Lane 9: CXCL12 + R54

Images captured with iBright 1500 by Thermo Fisher Scientific

Figure 4
Rac1 IGROV1

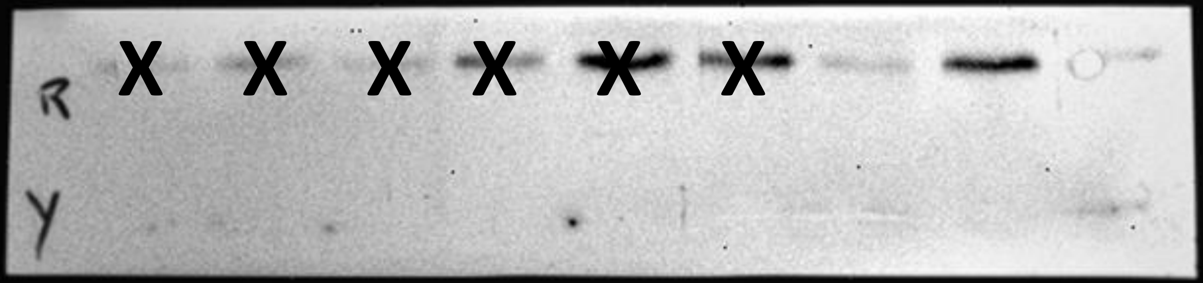

Mw ladder: Y=yellow 17 kDa, R=Red 22 KDa
Lane 7: UNTREATED
Lane 8: CXCL12
Lane 9: CXCL12 + R54

Images captured with iBright 1500 by Thermo Fisher Scientific

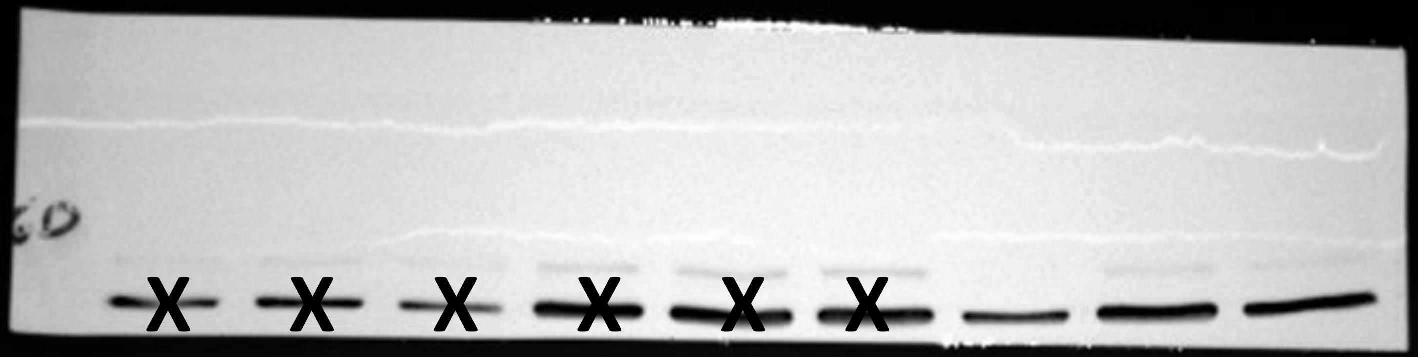

Figure 4
Tubulin IGROV1

Mw ladder
Lane 7: UNTREATED
Lane 8: CXCL12
Lane 9: CXCL12 + R54

Images captured with iBright 1500 by Thermo Fisher Scientific

Figure S3 B
E-Cadherin CAOV3 and IGROV1

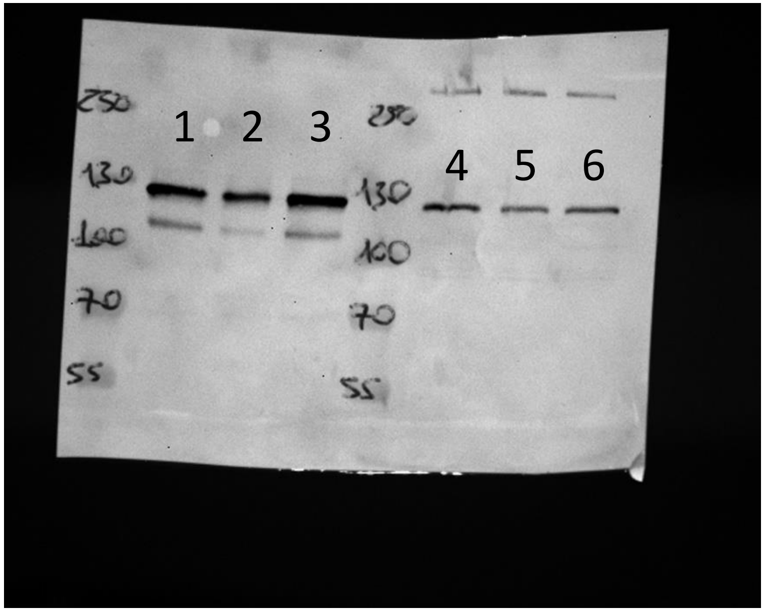

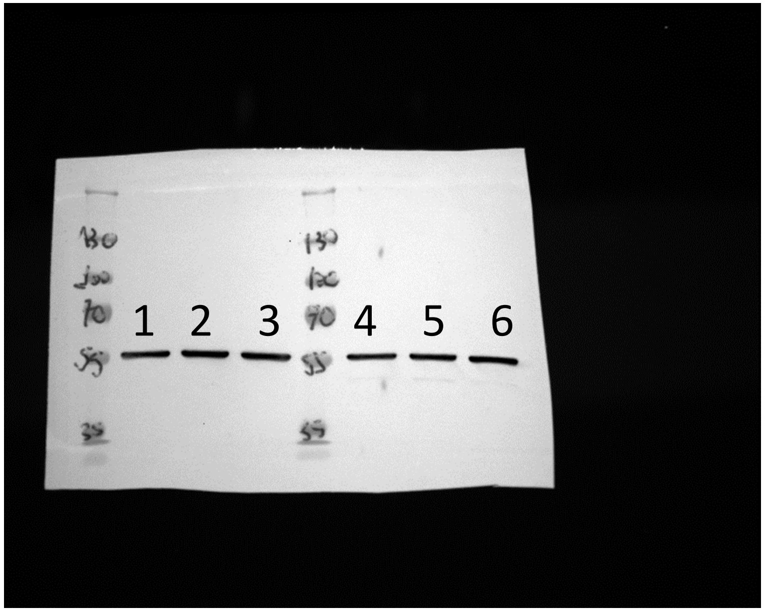

Mw
Lane 1: CAOV3 CTR
Lane 2: CAOV3 CXCL12
Lane 3: CAOV3 CXCL12 + R54
MW
Lane 4: IGROV1 CTR
Lane 5: IGROV1 CXCL12
Lane 6: IGROV1 CXCL12 + R54

Images captured with iBright 1500 by Thermo Fisher Scientific

Figure S4 B
beta-catenin IGROV1 KO

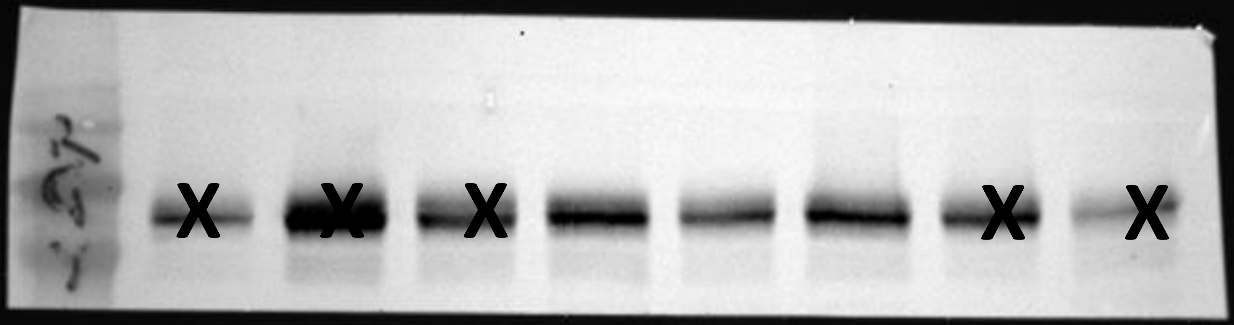

Mw ladder: R=Red 150 kDa, G=Green 102 KDa, Y=Yellow 76 KDa
Lane 4: UNTREATED
Lane 5: CXCL12
Lane 6: CXCL12 + R54

Images captured with iBright 1500 by Thermo Fisher Scientific

Figure S4 B
Vimentin IGROV1

Mw ladder: P=purple 52 KDa, B=Blue 38 KDa
Lane 4: UNTREATED
Lane 5: CXCL12
Lane 6: CXCL12 + R54

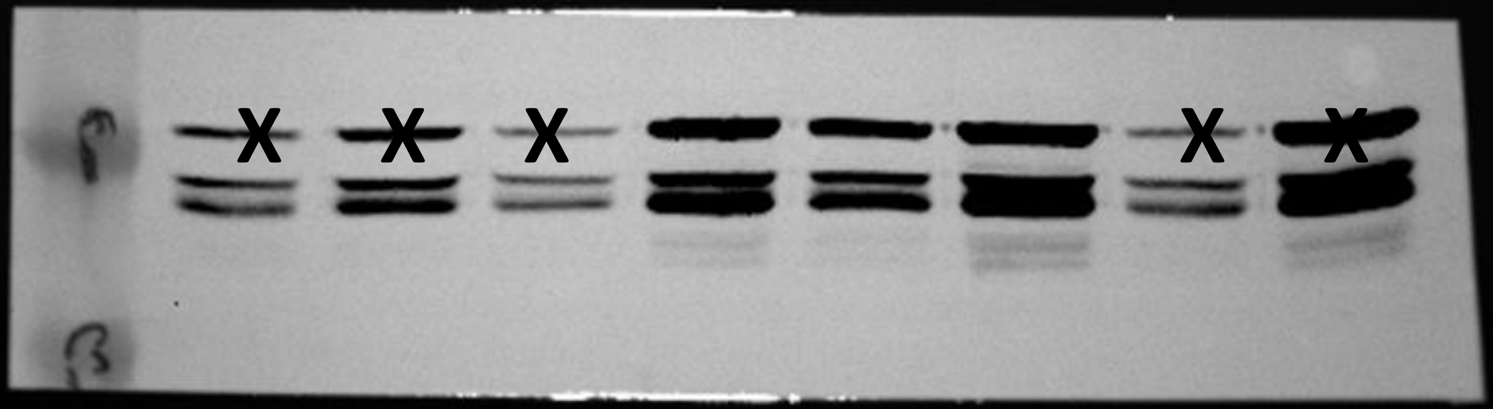

Images captured with iBright 1500 by Thermo Fisher Scientific

Figure S4 C

pERK IGROV1 KO

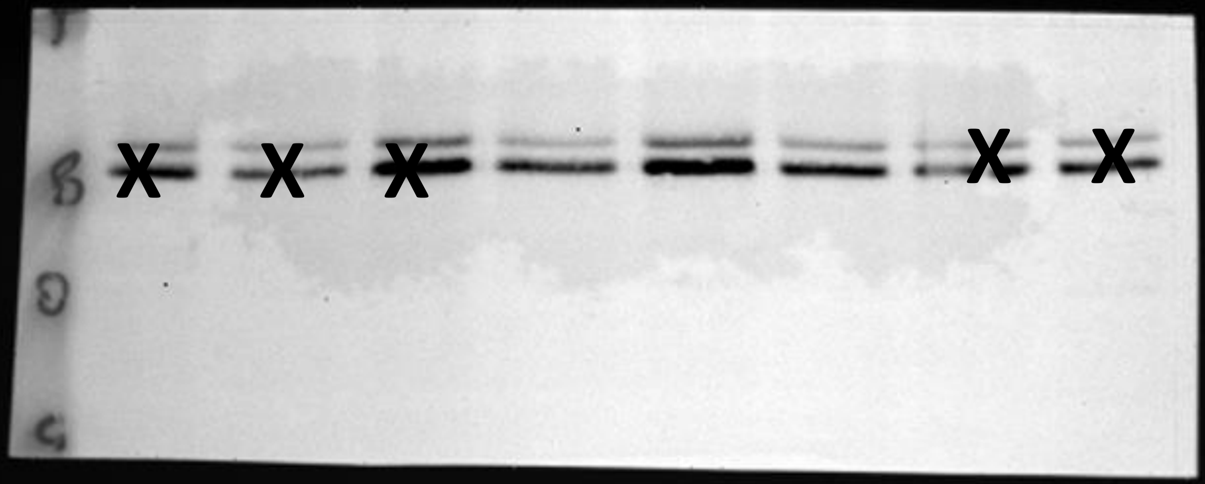

Mw ladder: B=Blue 38 KDa, O=Orange 31 KDa

Lane 4: UNTREATED

Lane 5: CXCL12

Lane 6: CXCL12 + R54

Images captured with iBright 1500 by Thermo Fisher Scientific

Figure S4 C
ERK tot IGROV1 KO

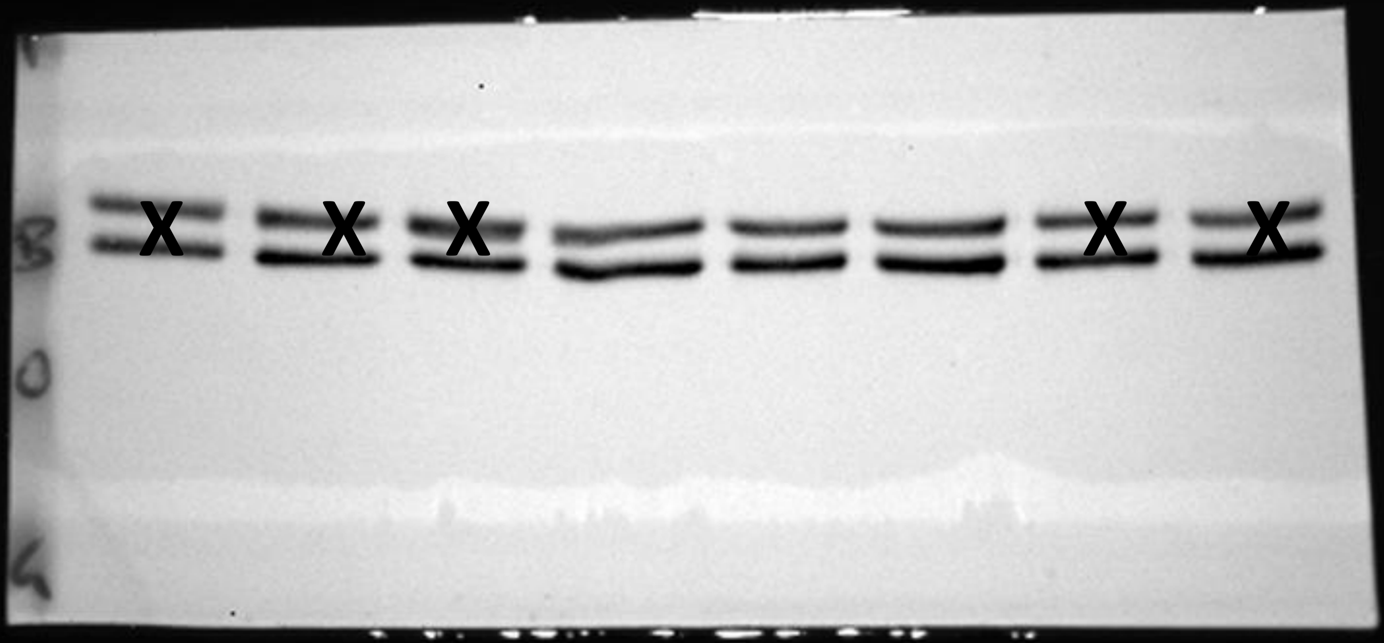

Mw ladder: B=Blue 38 KDa, O=Orange 31 KDa
Lane 4: UNTREATED
Lane 5: CXCL12
Lane 6: CXCL12 + R54

Images captured with iBright 1500 by Thermo Fisher Scientific

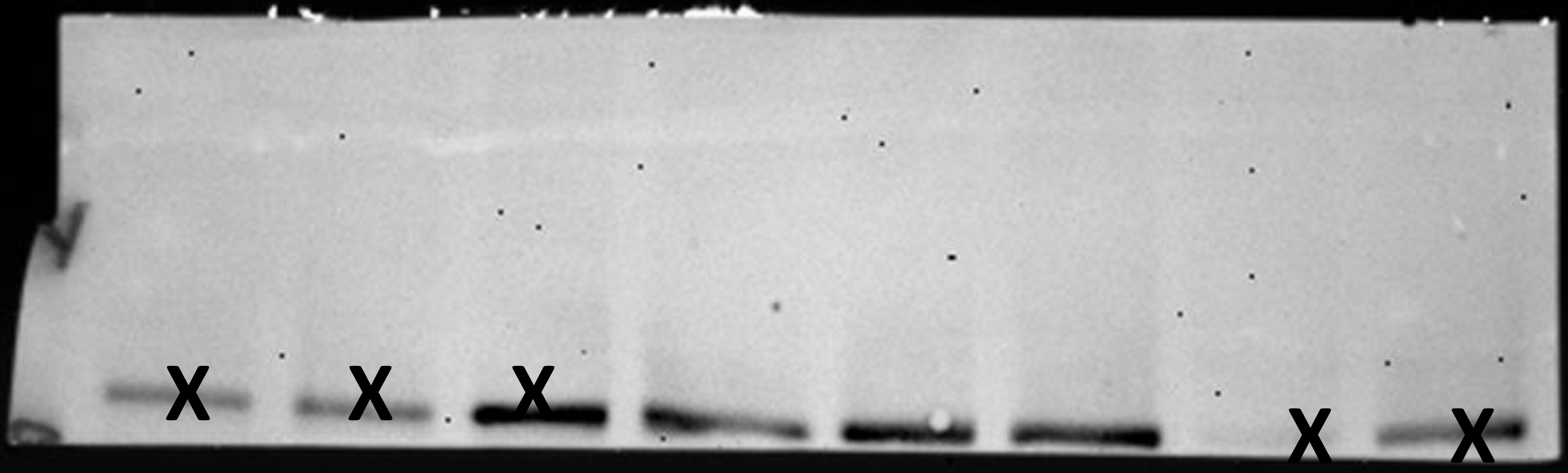

Figure S4 C
pAkt IGROV1 KO

Mw ladder: Y=yellow 76 KDa, P=purple 52 KDa
Lane 4: UNTREATED
Lane 5: CXCL12
Lane 6: CXCL12 + R54

Images captured with iBright 1500 by Thermo Fisher Scientific

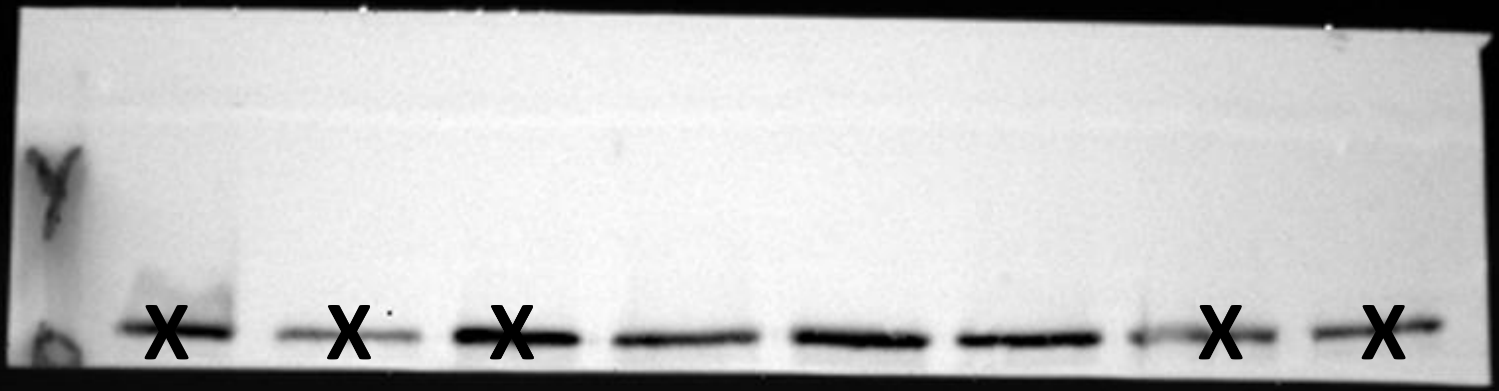

Figure S4 C
Akt tot IGROV1 KO

Mw ladder: Y=yellow 76 KDa, P=purple 52 KDa
Lane 4: UNTREATED
Lane 5: CXCL12
Lane 6: CXCL12 + R54

Images captured with iBright 1500 by Thermo Fisher Scientific

Figure S4 C

p-p38 IGROV1 KO

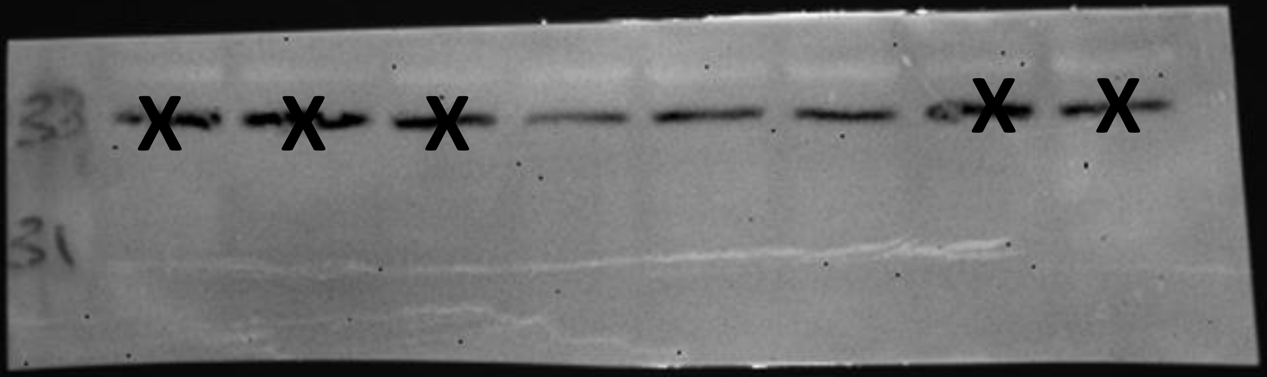

Mw ladder

Lane 4: UNTREATED

Lane 5: CXCL12

Lane 6: CXCL12 + R54

Images captured with iBright 1500 by Thermo Fisher Scientific

Figure S4 C
p38 IGROV1 KO

Mw ladder
Lane 4: UNTREATED
Lane 5: CXCL12
Lane 6: CXCL12 + R54

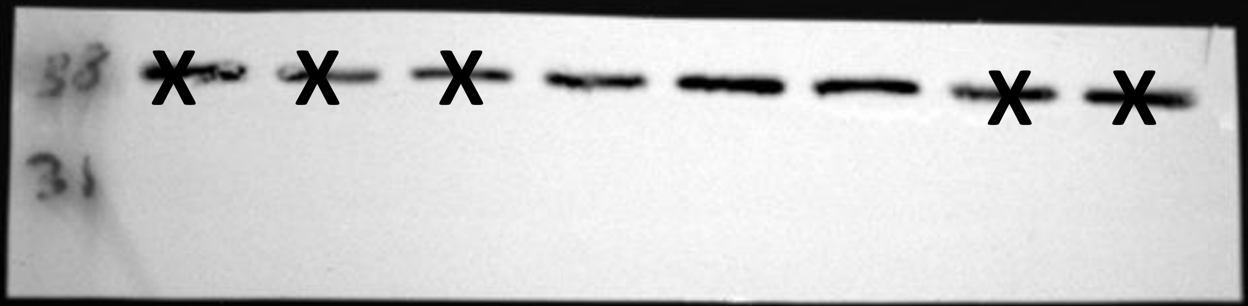

Images captured with iBright 1500 by Thermo Fisher Scientific

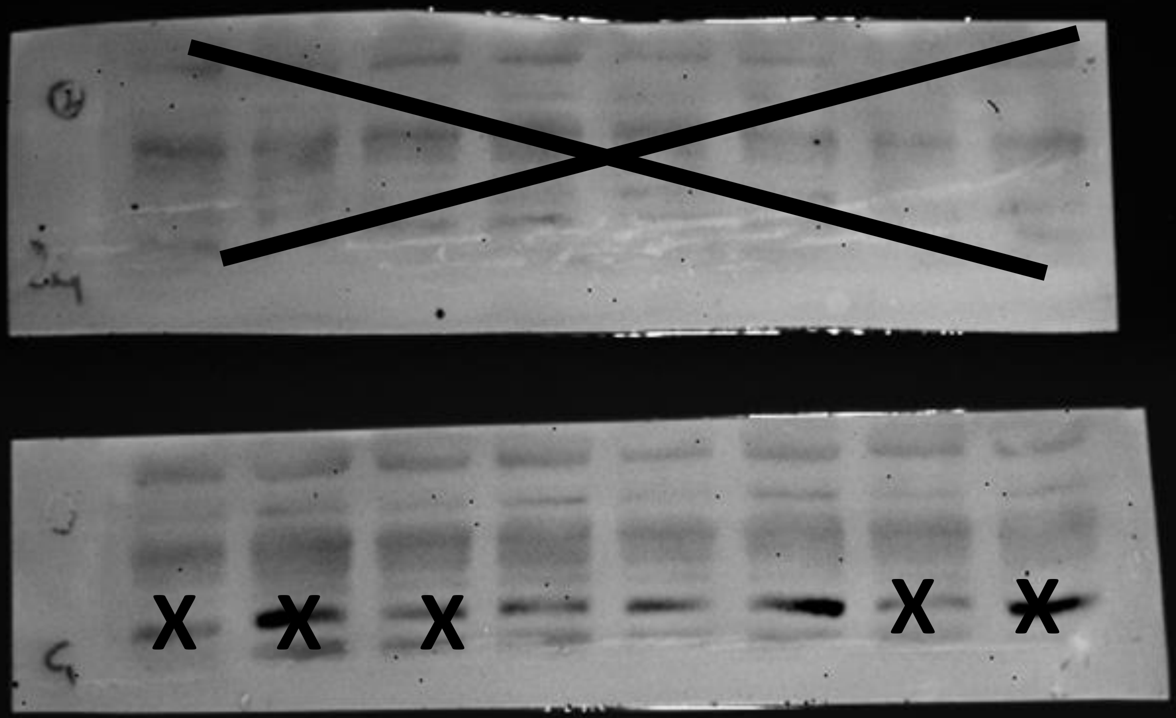

Figure S4 C
Rac1 IGROV1 KO (blot below)

Mw ladder: O=Orange 31 KDa, G=Green 24 KDa
Lane 4: UNTREATED
Lane 5: CXCL12
Lane 6: CXCL12 + R54

Figure S4 B e C
Tubulin IGROV1 KO

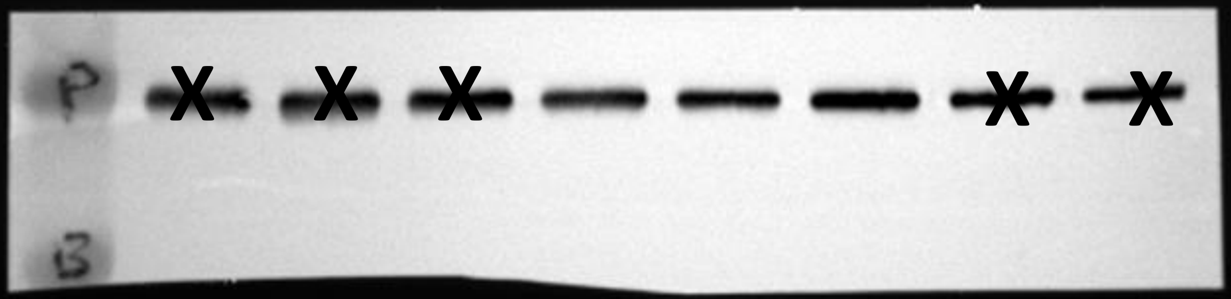

Mw ladder: P=purple 52 KDa, B=Blue 38 KDa
Lane 4: UNTREATED
Lane 5: CXCL12
Lane 6: CXCL12 + R54

Images captured with iBright 1500 by Thermo Fisher Scientific
